# Feasibility of Automated Image-Based Red Bone Marrow Dosimetry for [^177^Lu]Lu-PSMA Radiopharmaceutical Therapy of Metastatic Castration-Resistant Prostate Cancer

**DOI:** 10.3390/cancers17142313

**Published:** 2025-07-11

**Authors:** Mikhail Rumiantcev, Sandra Resch, Grigory Liubchenko, Gabriel Sheikh, Mathias Zacherl, Rudolf A. Werner, Sibylle I. Ziegler, Guido Böning, Astrid Delker

**Affiliations:** 1 Department of Nuclear Medicine, LMU University Hospital, LMU Munich, 81377 Munich, Germany; 2 The Russell H. Morgan Department of Radiology and Radiological Sciences, Division of Nuclear Medicine and Molecular Imaging, John Hopkins School of Medicine, Baltimore, MD 21287, USA; 3 Clinic for Nuclear Medicine, Hannover Medical School (MHH), 30625 Hannover, Germany

**Keywords:** mCRPC, radiopharmaceutical therapy, [^177^Lu]Lu-PSMA, red bone marrow, dosimetry

## Abstract

**Background/Objectives**: Red bone marrow irradiation is a major concern for patients with advanced prostate cancer undergoing [^177^Lu]Lu-PSMA therapy. However, low uptake in the red bone marrow and the presence of bone lesions complicate image-based red bone marrow dosimetry. This study aimed to investigate the general feasibility of image-based red bone marrow activity estimation for [^177^Lu]Lu-PSMA treatment and to develop a fully automated workflow for clinical implementation. **Methods**: In the first part of the study, 175 virtual patient phantoms with realistic ^177^Lu activity distributions were generated based on 639 pre-therapeutic [^18^F]F-PSMA-1007 PET/CT scans. The SIMIND Monte Carlo tool was used to simulate the ^177^Lu SPECT acquisitions (24 h post-injection (p.i.)), which were used to assess the uncertainty of red bone marrow activity estimation. In the second part, red bone marrow self- and cross-absorbed doses were estimated for four therapy cycles of 20 patients. **Results**: The simulation study shows a significant overestimation of activity in skeletal sites with bone lesions, with median recovery coefficients (RCs) across all phantoms yielding a median of 225% (range: 106–1015%). In contrast, the median RCs were markedly lower in skeletal sites neighboring or distant to lesion-carrying sites (105% [72–163%] and 107% [77–130%], respectively). The median total absorbed dose to the red bone marrow was 20.8 mGy/GBq (range: 5.6–297.9 mGy/GBq). Median blood levels decreased with an increasing median cumulative total absorbed dose. **Conclusions**: Reliable estimation of activity concentration in skeletal sites without bone lesion infiltration has been shown to be feasible. Based on this finding, an automated workflow for routine image-based red bone marrow dosimetry was developed.

## Simple Summary

Hematologic toxicity is a major concern for patients with metastatic castration-resistant prostate cancer (mCRPC) who undergo [^177^Lu]Lu-PSMA therapy, which can expose the red bone marrow to radiation. Reliable image-based red bone marrow dosimetry for these patients is challenged by low physiological uptake in the red bone marrow and high activity in adjacent bone metastases. This study aimed to evaluate the feasibility of image-based red bone marrow activity quantification in the context of [^177^Lu]Lu-PSMA therapy. Using a large cohort of virtual patient phantoms derived from [^18^F]F-PSMA-1007 PET/CT scans, we simulated ^177^Lu SPECT imaging and investigated the accuracy of activity quantification in the red bone marrow. Furthermore, we developed a fully automated workflow for routine image-based red bone marrow dosimetry to allow for individualized monitoring of hematologic toxicity in clinical practice.

## 1. Introduction

Metastatic castration-resistant prostate cancer (mCRPC) is an advanced stage of prostate cancer, which is a significant contributor to cancer-related mortality among men worldwide. In 2020, it was estimated that approximately 375,304 men died from prostate cancer, making it the fifth leading cause of cancer death among men globally [1]. Over the past decade, radioligand therapy (RLT) using [^177^Lu]Lu-PSMA has emerged as a promising treatment option for patients with mCRPC [2,3]. A potential limiting factor associated with [^177^Lu]Lu-PSMA therapy is bone marrow suppression, which can result in anaemia, leukopenia, and thrombocytopenia [4]. Since red bone marrow is the primary site of haematopoiesis, its irradiation during radionuclide therapy is a major safety consideration. Therefore, accurate dosimetry of the red bone marrow would be desired to optimize treatment efficiency while minimizing toxicity.

Dosimetric approaches have already been proposed for red bone marrow dose assessment in patients with neuroendocrine tumors (NET) treated with [^177^Lu]Lu-DOTATATE [5] and in patients with metastatic hormone-sensitive prostate cancer (mHSPC) receiving [^177^Lu]Lu-PSMA-617 [6]. However, there remains a particular need for dedicated approaches in mCRPC, where the frequent presence of bone metastases poses additional challenges for red bone marrow dosimetry.

According to the European Association of Nuclear Medicine (EANM) Dosimetry Committee guidelines for bone marrow dosimetry [7], absorbed dose to the red bone marrow can be estimated from blood samples as a surrogate or based on quantitative imaging. The blood-based approach is well established, but it requires repeated blood sampling, which imposes an additional burden on both patients and clinical staff. More importantly, as stated in the guidelines, the blood-based method is only valid when there is no specific uptake of the radiopharmaceutical in bone or bone marrow cells. In contrast, quantitative imaging provides a direct estimate of the total activity present in the bone marrow, including the activity in the bone marrow cells, the activity in the blood cells present in the bone marrow, and the activity freely circulating in the bone marrow [7]. Since quantitative SPECT imaging is routinely acquired in most [^177^Lu]Lu-PSMA therapy protocols, it offers a practical alternative for estimating the red bone marrow absorbed dose. Thus, leveraging SPECT imaging data, as proposed in our automated workflow, may offer a scalable and clinically feasible alternative to blood-based red bone marrow dosimetry in clinical practice. When estimating the absorbed dose to the red bone marrow using quantitative imaging, several challenges should be considered. First, the exact location of red bone marrow, being heterogeneously distributed on the micrometre scale, is not directly accessible with clinical imaging devices due to their limited spatial resolution. Red bone marrow uptake is typically inferred [5,6] from the uptake in skeletal regions, assuming a specific volume fraction of red bone marrow within bones. Accurate image-based estimation of the activity in the red bone marrow after [^177^Lu]Lu-PSMA therapy is further challenged by low skeletal uptake. In particular, the spatial resolution of clinical SPECT imaging is insufficient to clearly separate low-uptake red bone marrow regions from adjacent high-uptake bone lesions, which may lead to inaccurate activity quantification. The limited spatial resolution of SPECT imaging results in activity spill-over, which can lead to an underestimation of the red bone marrow uptake, but also to an overestimation of the red bone marrow uptake in the presence of bone metastases.

Different spill-over reduction techniques, often referred to as partial volume correction (PVC) techniques, were utilized to improve the activity quantification in [^177^Lu]Lu-PSMA SPECT images. Liu et al. [8] investigated the impact of recovery coefficient (RC)-based, iterative deconvolution-based reblurred van Cittert and anatomy-based iterative Yang methods on activity estimation in a geometrical phantom containing spheres of different diameters. They also evaluated these methods for the kidneys and lesions based on different realizations of the activity distribution in the XCAT phantom and real patient data. Leube et al. [9] proposed a deep-learning-based PVC method for quantitative [^177^Lu]Lu SPECT imaging and analysed its performance based on measurements of different phantoms (sphere, ellipsoid, renal cortex, kidney, spleen). In a meeting report by Lu et al. [10], PVC using the reblurred van Cittert method was applied to [^177^Lu]Lu-PSMA-617 SPECT images when performing image-based bone marrow dosimetry, resulting in lower bone marrow absorbed doses. Marquis et al. [11,12] developed MIRDpvc, an RC-based PVC tool that characterizes PET and SPECT spatial resolution using measured RCs of a NEMA phantom, which is then used to calculate corrected activity estimates.

Recent studies by Grob et al. [6] and Lu et al. [13] focused on image-based bone marrow dosimetry for patients receiving [^177^Lu]Lu-PSMA-617. In the first study, Grob et al. performed volumetric image-based bone marrow dosimetry by drawing spherical VOIs in the lumbar and lower thoracic vertebrae and compared it with the blood-based method. In the second study, an automatic image-based bone marrow segmentation method was developed and bone marrow absorbed dose maps were calculated using simulated Voxel-S-value (VSV) kernels.

In this study, we created a virtual patient phantom dataset with realistic, PET-derived distributions of bone metastases. This virtual phantom dataset was incorporated into a dedicated SPECT simulation workflow, which would allow us to systematically investigate the uncertainty in ^177^Lu activity estimation within the red bone marrow compartment and study the influence of spill-over reduction techniques in the presence of bone metastases. According to the EANM Dosimetry Committee guidelines for bone marrow dosimetry [7], there are three groups of possible contributors to the total absorbed dose to the red bone marrow: (i) self-absorbed dose due to activity in the red bone marrow, which can be divided into contributions from activity in the red bone marrow cells, blood cells and the extracellular fluid in the red bone marrow; (ii) cross-absorbed dose due to activity uptake in bone tissue; (iii) cross-absorbed dose due to activity uptake in major accumulating organs and the remainder of the body. The results from the simulation study served as the foundation for the subsequent implementation of a fully automated dosimetric workflow for the post-therapeutic estimation of self- and cross-absorbed dose to the red bone marrow to investigate and support the monitoring of hematologic toxicity in patients with mCRPC undergoing [^177^Lu]Lu-PSMA therapy.

## 2. Materials and Methods

### 2.1. Virtual Phantom Study

#### 2.1.1. Simulation and Reconstruction

**Figure 1:**
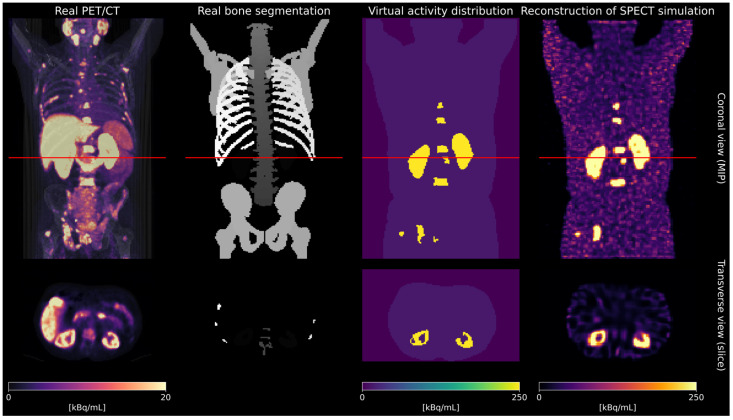
Dataset creation workflow. The red lines in the coronal view indicate the position of the transverse slices shown in the second row.

To define virtual patient phantoms with a realistic distribution of bone metastases, 639 pre-therapeutic [^18^F]F-PSMA-1007 PET/CT scans were analyzed. CT images were segmented using TotalSegmentator [14]. Binary masks for kidneys and bone lesions were generated using Otsu thresholding [15]. Bone lesions with an individual volume less than 1 mL were excluded. Based on a threshold of total bone lesion volume greater than 10 mL, the final database comprised 175 virtual patient phantoms. SPECT projection data were simulated using SIMIND (Version 7.0.3) [16]. The activity concentrations for the activity maps for SIMIND were derived from real SPECT images acquired 24 h post-injection (p.i.) for five patients treated with 7.4 GBq [^177^Lu]Lu-PSMA-I&T (mean ratio bone lesions–kidneys–background: 40:20:1). The simulation parameters were selected to mimic a clinical SPECT acquisition of patients undergoing [^177^Lu]Lu-PSMA therapy at our department (Siemens Symbia SPECT scanner (3/8” crystal thickness), Siemens Healthineers, Erlangen, Germany). An in-house MAP-OSEM algorithm (Bayesian weight 0.001) with attenuation correction, triple-energy-window scatter correction, and Gaussian resolution modeling enabled was used to reconstruct the SPECT images based on the simulated SPECT projection data. The workflow for creating the dataset is illustrated in Figure 1. Full simulation and reconstruction details are provided in the Supplementary Materials.

#### 2.1.2. Evaluation

The created dataset was employed to perform a RC analysis for VOIs in skeletal sites with and without bone lesions. The goal was to investigate the uncertainty of the activity concentration estimation in skeletal sites containing red bone marrow. The RC was calculated as

(1) $$RC=\frac{A_{recon}}{A_{reference}}\times 100\%,$$

where $A_{recon}$ and $A_{reference}$ are the reconstructed and the reference activity within a considered VOI, respectively.

**Figure 2:**
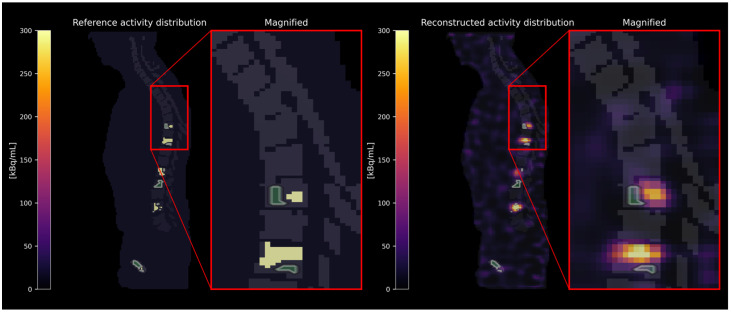
Exemplary volume of interest (VOI) selection in skeletal sites with bone lesions. The VOIs are depicted in green with white contour lines.

**Figure 3:**
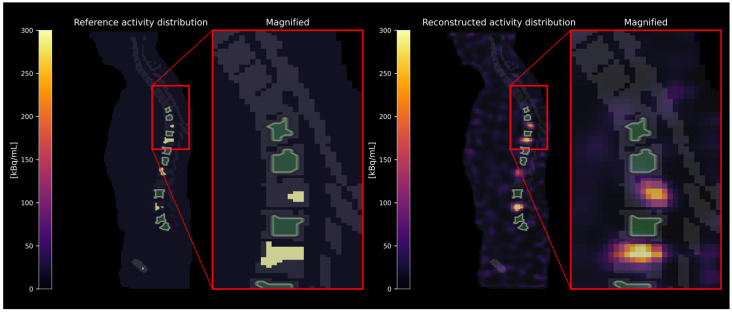
Exemplary VOI selection in neighboring skeletal sites without bone lesions but close to a skeletal site carrying a bone lesion. The VOIs are depicted in green with white contour lines.

**Figure 4:**
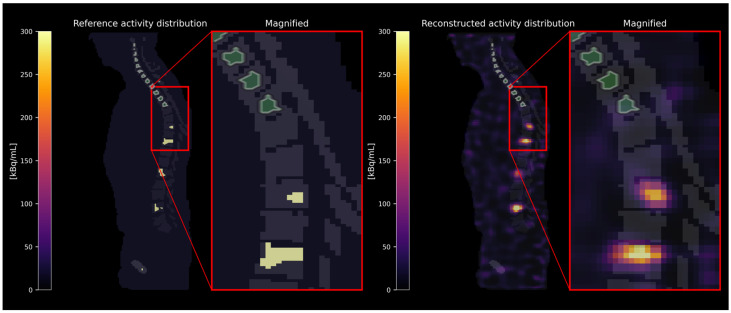
Exemplary VOI selection in distant skeletal sites without bone lesions in close proximity. The VOIs are depicted in green with white contour lines.

Activity concentrations were estimated in three different categories of skeletal sites: (i) skeletal sites (e.g., whole vertebrae) with bone lesions, as defined in the reference activity distribution (see Figure 2); (ii) skeletal sites carrying no bone lesions themselves but located adjacent to a skeletal site containing a bone lesion (see Figure 3); and (iii) distant skeletal sites without any bone lesions in close proximity (see Figure 4). The spatial proximity was determined by calculating the surface-to-surface distances between bone lesions and skeletal sites without bone lesions and defining a distance threshold of 30 mm. As the exact localization of the red bone marrow compartment is not accessible in [^177^Lu]Lu-PSMA SPECT/CT images, the red bone marrow compartment was assumed to be represented by specifically defined skeletal VOIs. These were defined (i) as eroded (binary erosion with 1 iteration, i.e., excluding the outer margins of the bone compartment to minimize inclusion of cortical bone) bone compartment VOIs excluding dilated (binary dilation with 1 iteration, i.e., expanding the bone lesion masks to account for potential spill-over) bone lesion VOIs or (ii) as eroded bone compartment VOIs only in case of the absence of bone lesions. We assumed that no red bone marrow is present within the bone lesions themselves [17]. Table 1 summarizes the VOI selection in different skeletal sites. The minimum volume of defined skeletal VOIs included in the analysis was set to 1 mL.

For the sake of completeness, the RC analysis was also performed for the bone lesions and the kidneys.

To assess inter-phantom variability in red bone marrow RCs, the median RC was calculated individually for each virtual phantom. For the entire cohort, the overall median and range of these individual median values are reported.

#### 2.1.3. Application of Spill-Over Reduction Techniques

Two different methods, which were established for molecular imaging, were investigated to compensate for the limited spatial resolution in SPECT imaging and thus the activity spill-over from bone lesions in the bone marrow compartment: (i) iterative Yang (IY) and (ii) Lucy–Richardson deconvolution (LR). Briefly, LR is an iterative deconvolution-based approach [18,19] that corrects for the blurring of the activity distribution caused by the imaging system’s point spread function (PSF). IY also considers the PSF but incorporates anatomical constraints (masks) [20,21]. We used the IY implementation provided by the PETPVC toolbox [22] and the LR implementation available in the Python (Version 3.11.0) package scikit-image (Version 0.23.2). The PSF was estimated based on a simulated and reconstructed NEMA IEC body phantom and the matched filter analysis presented in [23] (total activity of 1000 MBq; sphere-to-background ratio of 40:1, similar to the lesion-to-background ratio in the aforementioned prostate cancer patient cohort of Section 2.1.1). Briefly, the reference activity distribution (simulation input) was convolved with isotropic 3D Gaussian functions, where the standard deviation σ varied from 0.1 mm to 5.0 mm in 0.1 mm steps. The corresponding full-width-half-maximum (FWHM) was computed as $FWHM=2\sqrt{2\ln2}\cdot \sigma$ (corresponding FWHM range: 0.24 mm to 11.77 mm). The blurred reference activity distribution was then compared to the reconstructed activity distribution of the simulated NEMA phantom. The FWHM at which the minimum root-mean-square-error between the blurred reference activity distribution and the reconstructed activity distribution was reached was considered as the FWHM describing the system’s PSF.

When using IY, the masks for the kidneys and bone lesions were each defined in two different ways: (i) based on the ground truth masks, i.e., the activity maps used for the simulation; (ii) based on the masks created by applying Otsu thresholding to the reconstructed activity distribution. The latter shall represent the clinical situation, in which the ground truth lesion segmentation is usually not available.

For both, IY and LR, the optimal number of iterations $n_{iter}$ was estimated by varying the number of iterations and calculating the sphere RCs and the contrast-to-noise ratios (CNRs).

#### 2.1.4. Influence of Time per Projection, Poisson Noise, and Volume Threshold on RC Estimation in the Red Bone Marrow

In addition to the default acquisition regime (5 s per projection with Poisson noise applied), the influence of time per projection, Poisson noise, and VOI volume threshold (for the assumed red bone marrow VOI) on red bone marrow RCs was investigated. Specifically, the same simulated projection data was additionally processed according to two regimes: (i) setting the time per projection to 15 s instead of 5 s and applying Poisson noise; (ii) setting the time per projection to 15 s without applying Poisson noise. Furthermore, the impact of different VOI volume thresholds (1 mL, 5 mL, and 15 mL) on RC variability and accuracy was evaluated.

### 2.2. Patient Study

**Figure 5:**
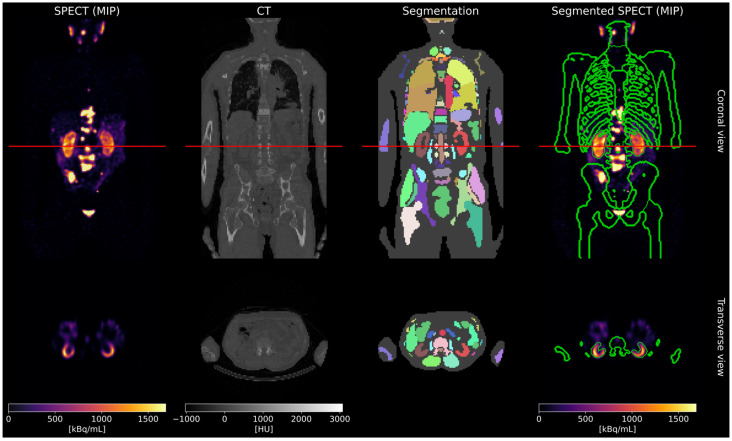
Real patient data processing. The red lines in the coronal view indicate the position of the transverse slices shown in the second row.

Based on the results from the virtual phantom study, an automated image-based workflow was developed to support the investigation and estimation of the red bone marrow self- and cross-absorbed doses in clinical post-therapeutic patient data. SPECT/CT scans acquired at 24, 48, and 72 h post administration of [^177^Lu]Lu-PSMA-I&T were used, and the acquisition parameters matched those applied in the simulation study (5 s per projection; 128 angles; 128 × 128 matrix). A visualization of the workflow is depicted in Figure 5. It incorporated CT segmentation using TotalSegmentator [14], CT-to-SPECT registration, bone lesion segmentation, estimation of VOI activity concentrations and fitting of time-activity curves to estimate the time-integrated activities (TIAs) and the absorbed doses. All steps, including bone lesion segmentation, were performed fully automatically as part of the reproducible workflow. The entire pipeline was implemented in a scripted manner, ensuring that repeated application to the same data yields identical results. Further details are specified in the following.

#### 2.2.1. Self-Absorbed Dose

To segment the bone lesions in the ^177^Lu SPECT images, an SUV threshold of 2 was first applied to all the bone compartments available in TotalSegmentator [14], followed by the Otsu thresholding method as conducted in the virtual phantom study. Skeletal VOIs were defined in skeletal sites without bone lesions (both neighboring and distant, as described in the virtual phantom study (methods 2 and 3)). All the activity present in a selected bone site was assumed to be located in the red bone marrow, assuming the absence of specific binding to bone tissues [2,24,25,26,27]. The red bone marrow mass required for dosimetry was estimated from the local volume fraction of red bone marrow based on [28] and the red bone marrow density of $\approx 1.03\;g/{cm}^{3}$ suggested by ICRP 89 [29]. As described in [28], the volume fraction of red bone marrow $f_{rbm}$ was calculated as

(2) $$f_{rbm}\;=\;f_{s}\;\times \;f_{m}\;\times \;c,$$

where $f_{s}$, $f_{m}$, and c denote the spongiosa volume fraction, the marrow volume fraction in spongiosa, and the cellularity, respectively. The red bone marrow S-value for self-absorbed dose was taken from OLINDA/EXM (Version 2.0) [30] with appropriate mass scaling. The TACs were fitted mono-exponentially. Only TACs for which the curve fit showed a coefficient of determination of $R^{2}>0.95$ were included in the analysis. The mean self-absorbed doses to the whole red bone marrow per patient and per cycle were calculated as mass-weighted mean values across all evaluated skeletal sites. The uncertainties of the red bone marrow self-absorbed doses per skeletal site were estimated based on the uncertainties of the estimated fit parameters using propagation of uncertainty. The uncertainties of the mean self-absorbed doses to the whole red bone marrow per patient and per cycle were estimated from the uncertainties of the red bone marrow self-absorbed doses per skeletal site and the uncertainties of the assumed red bone marrow masses per skeletal site across the scans of the respective cycle using propagation of uncertainty.

#### 2.2.2. Cross-Absorbed Dose

The cross-absorbed dose to the red bone marrow was estimated according to the EANM guideline [7], assuming contributions primarily from the kidneys and the remainder of the body. In this simplified approach, the contribution from the remainder of the body was estimated by subtracting the TIAs of the kidneys and the assumed red bone marrow from the total-body TIA. This means that lesions are implicitly included as part of the remainder of the body. Patient-specific S-values were derived by scaling standard OLINDA/EXM [30] phantom values based on individual organ and body masses. The corresponding formulas can be found in the Supplementary Materials.

## 3. Results

### 3.1. Virtual Phantom Study

The estimated median RCs for methods 1–3 as defined in Table 1, including the effect of spill-over reduction, time per projection, Poisson noise, and the VOI volume threshold are summarized in the Supplementary Materials (see Supplementary Table S1). According to the matched filter analysis, the FWHM of the PSF required for both IY and LR was estimated to be 8.3 mm. The optimal number of iterations for IY and LR was set to 5 and 10, respectively (Supplementary Figures S1–S6).

**Figure 6:**
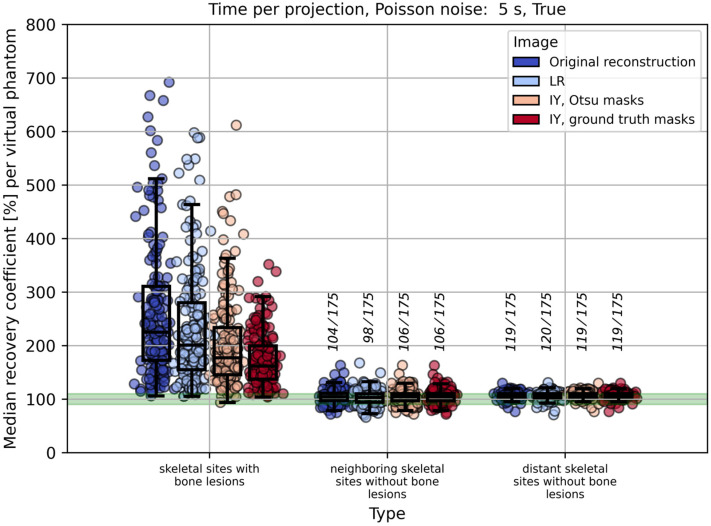
Median recovery coefficients (RCs) per virtual phantom for methods 1–3 (as defined in Table 1), evaluated for the default clinical regime (5 s per projection with Poisson noise applied). Results are shown with and without spill-over reduction. The minimum volume of analyzed skeletal VOIs is 1 mL. The green band indicates the RC range of 90–110%, provided for visual orientation. Vertical fractions show the number of virtual phantoms (out of 175) with median RCs within the green band.

Across all measurement times, noise settings and volume thresholds, a marked overestimation of RCs was observed in skeletal sites with bone lesions. IY with the ground truth masks for the bone lesions reduced the overestimation most effectively. IY with the Otsu-based lesions masks provided intermediate improvement, while LR deconvolution showed the least reduction among the tested methods. In contrast, in skeletal sites without bone lesions (neighboring and distant), the spill-over reduction techniques resulted in minimal or no changes compared to the original reconstruction. In these regions, the reported median RCs across all virtual phantoms remained close to 100%. Figure 6 illustrates this general trend for the default acquisition regime (5 s per projection, Poisson noise applied), which mimics the clinical protocol.

**Figure 7:**
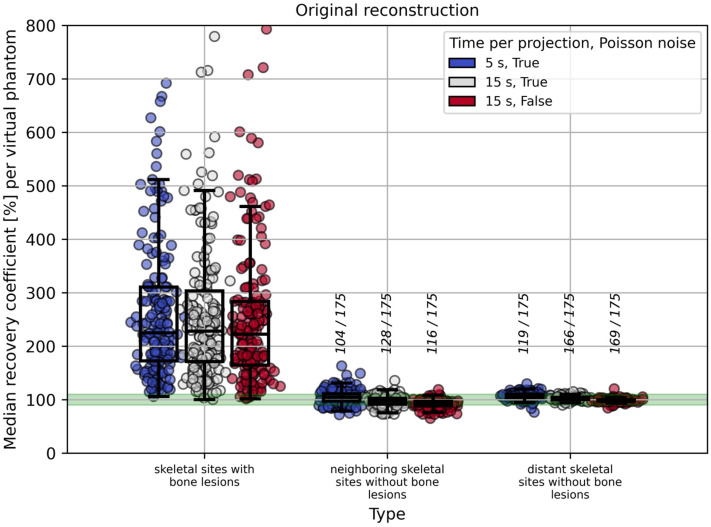
Median RCs per virtual phantom for methods 1–3 (as defined in Table 1) evaluated for time per projection of 5 s and 15 s, and in the presence or absence of Poisson noise. Results are based on the original reconstructed images. The minimum volume of analyzed skeletal VOIs is 1 mL. The green band indicates the RC range of 90–110%, provided for visual orientation. Vertical fractions show the number of virtual phantoms (out of 175) with median RCs within the green band.

In skeletal sites without bone lesions, increasing the time per projection from 5 s to 15 s and applying or omitting Poisson noise to the simulated data led to a decrease in RCs, whereas these changes had comparatively little effect on RC estimates in skeletal sites with bone lesions. Figure 7 shows this exemplarily for the original reconstructed images, i.e., without any spill-over reduction applied.

**Figure 8:**
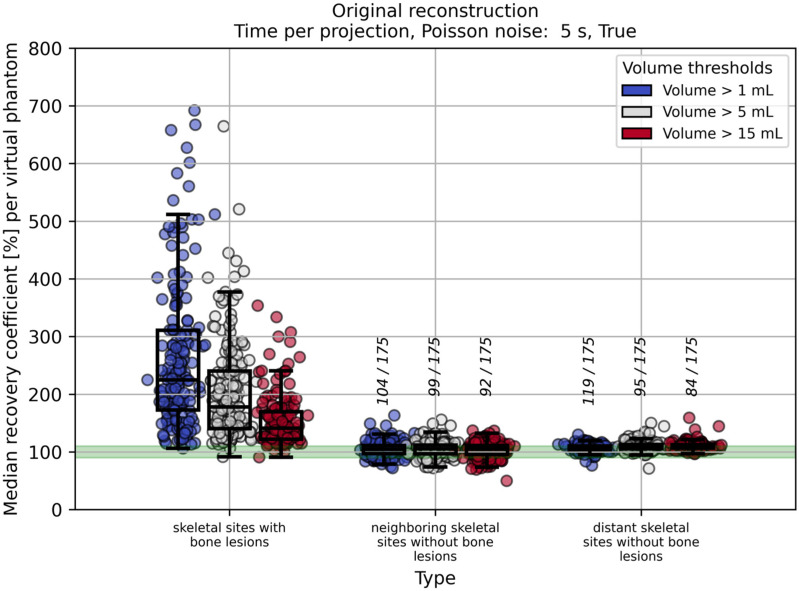
Median RCs per virtual phantom for methods 1–3 (as defined in Table 1) evaluated across different VOI volume thresholds. Results are shown for the default clinical regime (5 s per projection with Poisson noise) using the original reconstructed images. The green band indicates the RC range of 90–110%, provided for visual orientation. Vertical fractions show the number of virtual phantoms (out of 175) with median RCs within the green band.

Increasing the VOI volume threshold from 1 mL to 15 mL led to a clear decrease in activity overestimation for skeletal sites with bone lesions, while RCs in skeletal sites without bone lesions remained largely unaffected. Figure 8 demonstrates this effect for the default regime (5 s per projection with Poisson noise applied) using the original reconstructed images.

A comprehensive set of plots illustrating the effects of spill-over reduction techniques, time per projection, Poisson noise added or not, and the selected VOI volume thresholds on RC estimation is provided in the Supplementary Materials (see Supplementary Figures S7–S22). For the sake of completeness, the estimated RCs in the kidneys and bone lesions are also shown in the Supplementary Materials (see Supplementary Figure S23).

### 3.2. Patient Study

**Figure 9:**
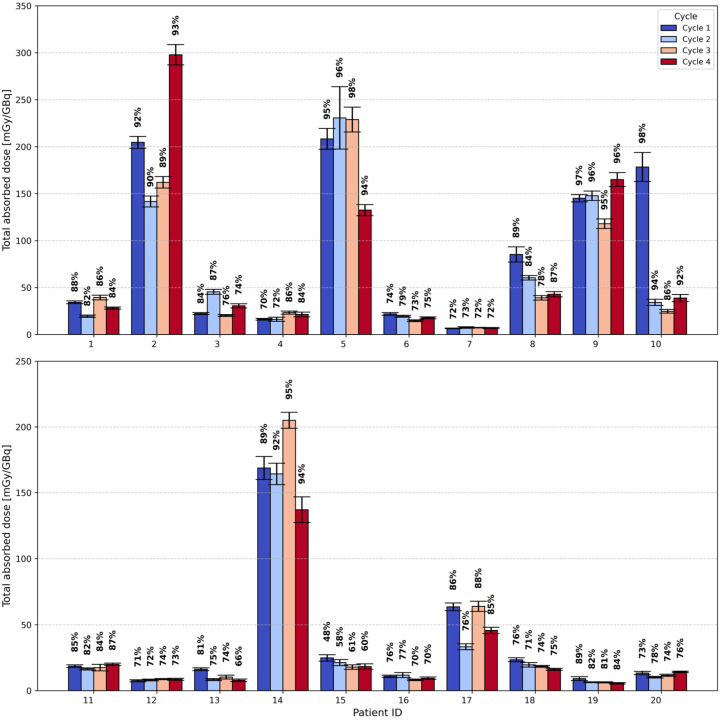
Estimated total absorbed doses to the red bone marrow per treatment cycle for 20 patients, each with four analyzed cycles. The relative contribution of the self-absorbed dose to the total absorbed dose is indicated above each bar.

Figure 9 provides the estimated total absorbed doses to the red bone marrow per cycle for 20 patients (7.4 GBq or 9 GBq, [^177^Lu]Lu-PSMA-I&T) as analyzed over four cycles. As can be seen in Figure 9, patient-specific red bone marrow dosimetry shows high variability. The median red bone marrow self-absorbed dose for the analyzed patients was 15.9 mGy/GBq (min: 4.7 mGy/GBq; max: 277.0 mGy/GBq), while the median cross-absorbed dose to the red bone marrow was estimated to be 4.3 mGy/GBq (min: 0.9 mGy/GBq; max: 20.9 mGy/GBq). The resulting median total absorbed dose to the red bone marrow was 20.8 mGy/GBq (min: 5.6 mGy/GBq; max: 297.9 mGy/GBq). The fraction of the self-absorbed dose to the red bone marrow relative to the total absorbed dose had a median of 82% (min: 48%, max: 98%), indicating that self-irradiation was overall the dominant contributor to red bone marrow absorbed dose in the studied cohort. Patients with IDs 2, 5, 8, 9, 10, 14, 17 presented a high bone lesion volume with elevated uptake, resulting in higher red bone marrow self-absorbed doses (median: 127.6 mGy/GBq; min: 21.4 mGy/GBq; max: 277.0 mGy/GBq) compared to the rest of the cohort (median: 11.2 mGy/GBq; min: 4.7 mGy/GBq; max: 39.4 mGy/GBq). The corresponding median total absorbed dose to the red bone marrow was 139.4 mGy/GBq (min: 24.8 mGy/GBq; max: 297.9 mGy/GBq) for these patients and 16.3 mGy/GBq (min: 5.6 mGy/GBq; max: 45.4 mGy/GBq) for the remaining patients. For comparison, the first-cycle SPECT images at 24 h p.i. for exemplary patients with high and low tumor volume can be found in the Supplementary Materials (see Supplementary Figures S24 and S25). No trend was observed for the self- and cross-absorbed dose to the red bone marrow over four cycles.

**Figure 10:**
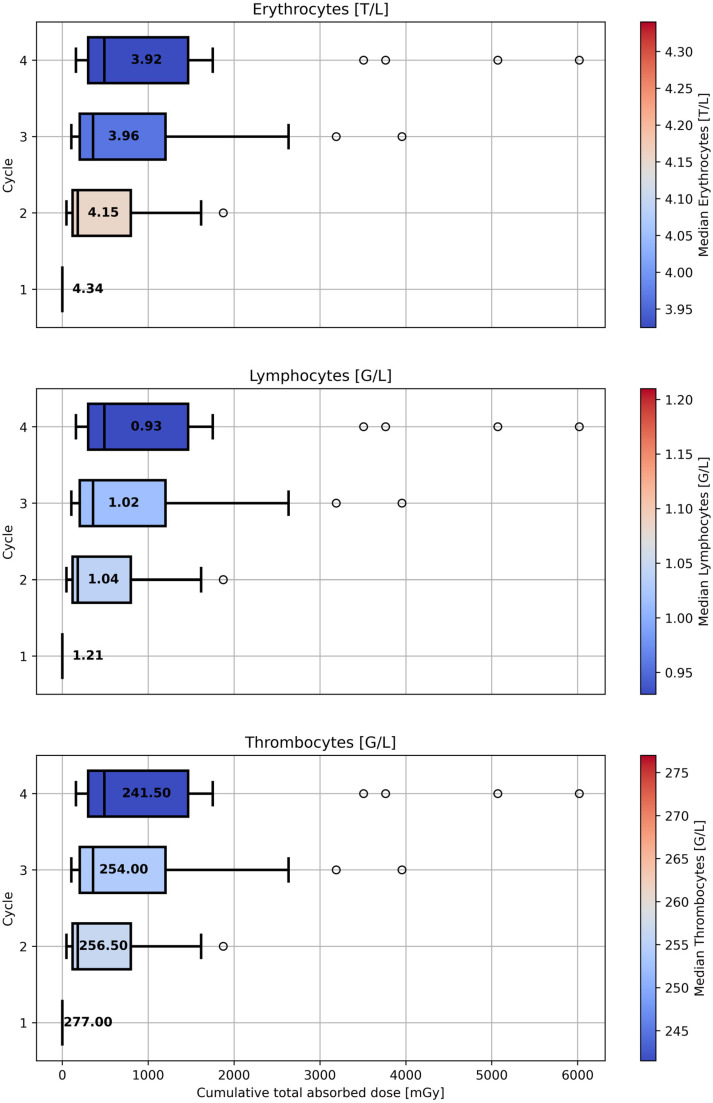
Median blood levels of erythrocytes, lymphocytes, and thrombocytes prior to each injection shown in relation to the cumulative total absorbed doses to the red bone marrow up to the respective cycle. Box colors indicate the cycle-specific median blood parameter values, which are also annotated numerically. Units: G/L = $10^{9}$ cells per liter; T/L = $10^{12}$ cells per liter.

**Figure 11:**
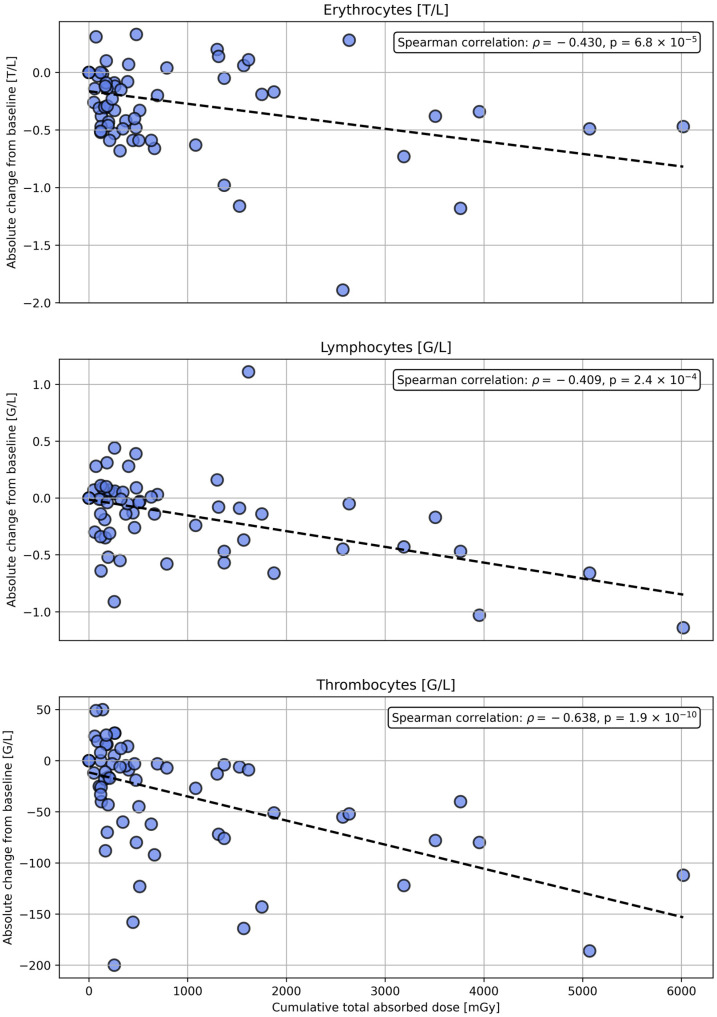
Correlation between cumulative total absorbed dose to the red bone marrow and absolute change in blood levels of erythrocytes, lymphocytes, and thrombocytes (measured prior to each injection) from baseline. Dashed black lines indicate the linear regression fit (shown for illustrative purpose). Spearman’s rank correlation coefficient (ρ) and corresponding *p*-value are provided to assess the strength and significance of monotonic trends. Units: G/L = $10^{9}$ cells per liter; T/L = $10^{12}$ cells per liter.

Blood levels of erythrocytes, lymphocytes, and thrombocytes prior to each injection across four therapy cycles are shown in the Supplementary Materials (see Supplementary Figure S26). The median blood levels of erythrocytes, lymphocytes, and thrombocytes, measured prior to each injection, are illustrated in Figure 10 in relation to the estimated cumulative total absorbed doses to the red bone marrow up to the corresponding cycle. The general trend shows a decrease in median blood levels with increasing median cumulative total absorbed dose. The median cumulative total absorbed dose to the red bone marrow prior to cycles 1–4 was 0 mGy, 179.8 mGy (min: 49.1 mGy; max: 1873.6 mGy), 357.8 mGy (min: 104.5 mGy; max: 3954.2 mGy), and 490.5 mGy (min: 159.0 mGy; max: 6018.3 mGy), respectively. In addition, for each patient and cycle, the absolute change in blood levels from baseline (prior to cycle 1) was calculated and plotted against the cumulative total absorbed dose to the red bone marrow up to that cycle. Figure 11 shows the correlation between the cumulative total absorbed dose to the red bone marrow and the absolute change in blood levels from baseline. A statistically significant inverse correlation was observed for all three parameters, with the strongest effect seen for thrombocytes, followed by erythrocytes and lymphocytes. These results support the observed trend of decreasing blood levels with the increasing of the cumulative total absorbed dose to the red bone marrow.

## 4. Discussion

Image-based red bone marrow dosimetry in patients receiving [^177^Lu]Lu-PSMA therapy poses significant challenges due to the low activity concentration in the red bone marrow and the presence of bone metastases, which can contaminate surrounding red bone marrow regions through a spill-over effect. To address these challenges, we first investigated the uncertainty and general feasibility of red bone marrow activity estimation using a dedicated simulation dataset. Based on the insights gained, we developed a dosimetric workflow aimed at enabling automated practical red bone marrow dosimetry for routine clinical use.

To evaluate the accuracy of image-based red bone marrow activity estimation, we analyzed RCs in three distinct VOI categories (methods 1, 2, and 3 from Table 1). Method 1, which relies on skeletal sites with bone lesions, led to considerable overestimation and is therefore less suitable for red bone marrow activity estimation. Methods 2 and 3, both considering skeletal sites without bone lesions, were found to be suitable for activity estimation in the red bone marrow. It must be noted that in patients with a high tumor burden, only a few—or in some cases no—appropriate VOIs may be available according to methods 2 or 3. In such cases, method 1 may need to be considered; however, spill-over reduction techniques should then be applied (see Figure 6), and a sufficiently large VOI volume threshold should be used to reduce the impact of local activity overestimation (see Figure 8). For method 1, increasing the time per projection had a minor effect on RC estimation compared to the influence of spill-over reduction and the VOI volume threshold. In contrast, for methods 2 and 3, a decrease in RCs was observed with longer acquisition times and when Poisson noise was omitted (see Figure 7 and Supplementary Table S1).

The high variability in RCs observed in skeletal VOIs for all methods is due to several reasons. For method 1, the variability in RCs is primarily attributed to the extent to which bone lesions of different shapes and volumes contaminate the regions surrounding them. For the VOIs from method 1, the VOI volume plays a crucial role. Setting the volume threshold for these VOIs from 1 mL to 5 mL or even 15 mL leads to a lower overall median RC and reduces the variability of the estimated RCs (see Supplementary Table S1 and Figure 8). Further, for all methods, the variability in RCs is partly related to Poisson noise. As can be seen in Supplementary Table S1 and Figure 7, setting the time per projection to 15 s instead of 5 s and disabling Poisson noise partly leads to a slightly lower variability of the estimated RCs. Even in the absence of Poisson noise, RC variability may arise due to fluctuations in the reconstructed images, which are inherent to the reconstruction algorithm, and from shape-dependent variations in recovery. It should be noted that the over- or underestimation of RCs in skeletal sites without bone lesions—calculated per virtual phantom—may also be influenced by scatter correction; however, investigation of this effect was not part of the present study.

A potential limitation of applying methods like IY or LR for spill-over reduction is related to the system’s PSF, which describes the blurring effect in SPECT images. Although resolution modeling during image reconstruction aims to correct for this blurring, the correction may not be perfect. The remaining blur is accounted for by assuming a specific PSF when applying spill-over reduction techniques. In our study, we used a simplified model of the PSF: an isotropic 3D Gaussian function. The accuracy of the spill-over reduction, and thus of the activity quantification and dosimetry, depends on how well this assumed PSF represents the actual system behavior.

As already mentioned in Section 2, the precise localization of the red bone marrow compartment is generally not accessible through [^177^Lu]Lu-PSMA SPECT imaging. This is a fundamental limitation of image-based red bone marrow dosimetry. In the simulation study, the skeletal VOIs were defined to potentially include the red bone marrow; therefore, by estimating the RCs in the defined skeletal VOIs, it is possible to investigate the effect of over- or underestimation of activity in the red bone marrow.

To summarize, based on a dataset of 175 virtual patient phantoms mimicking real patients who undergo [^177^Lu]Lu-PSMA therapy, it was shown that activity estimation in skeletal sites without bone lesions (methods 2 or 3) is feasible, considering the low activity concentration in these VOIs (a few tens of kBq/mL). As can be seen in Figure 6, for methods 2 and 3, the number of virtual phantoms (out of 175) with median RCs within the range 90–110% was 104 and 119, respectively.

Based on the methodological findings of the simulation study, image-based red bone marrow dosimetry was performed for 20 patients with mCRPC who received four cycles of treatment with 7.4 GBq or 9 GBq of [^177^Lu]Lu-PSMA-I&T.

The estimated red bone marrow absorbed doses are within a comparable range to those published previously. Beauregard et al. [31] reported a median absorbed dose to the bone marrow of 29 mGy/GBq (range: 3–776 mGy/GBq) using VOIs drawn in the L1 to L4 vertebrae for the first cycle treatment of 27 patients with mCRPC (7.0–7.6 GBq [^177^Lu]Lu-PSMA-I&T). Gosewisch et al. [17] estimated the median bone marrow absorbed dose to range between 11 and 130 mGy/GBq, depending on the dosimetry method used (3D Monte Carlo bone marrow dosimetry or via S-value dosimetry), based on 11 cycles of [^177^Lu]Lu-PSMA-617 therapy in 10 patients with mCRPC. In a recent publication by Grob et al. [6], the authors estimated the mean bone marrow absorbed dose to be 25.4 ± 8.7 mGy/GBq based on eight patients with mHSPC with two cycles (3 and 6 GBq) of [^177^Lu]Lu-PSMA-617 therapy by drawing 1.5 cm diameter spheres in the lowest 10 vertebrae. They compared this image-based approach with the blood-based method, which yielded a mean bone marrow absorbed dose of 17.2 ± 3.4 mGy/GBq. The differences between the aforementioned studies and the values reported in this study may arise due to differences in the patient cohorts, particularly with regard to the tumor burden, VOI positioning, or the methodology of absorbed dose calculation.

The main limitation of our dosimetry workflow is that the red bone marrow activity is inferred from macroscopic VOIs rather than directly imaged. In our approach, the activity in the red bone marrow is approximated by the total activity in skeletal sites without segmented bone lesions, which could lead to an overestimation of the self-absorbed dose to the red bone marrow. The reason for this approach is that red bone marrow is distributed on a micro-meter scale, which is below the spatial resolution of clinical imaging devices. The total red bone marrow mass per skeletal site was assessed according to the phantom used in [28], which is based on the data provided in ICRP 89 [29] and represents the average bone composition in a reference human. Recent studies have demonstrated that patient-specific assessment of bone marrow composition is achievable using magnetic resonance imaging (MRI) or dual-energy CT. Salas-Ramirez et al. [32] applied a two-point Dixon MRI sequence to quantify the fat fraction in lumbar vertebrae, revealing considerable inter-patient variability and emphasizing the relevance of individual bone marrow composition for red bone marrow dosimetry. In parallel, a dual-energy quantitative CT (DEQCT) method was introduced by Salas-Ramirez et al. [33], enabling estimation of patient-specific volume fractions of red marrow, yellow marrow, and bone mineral in the spongiosa through post-reconstruction material decomposition on a clinical (SPECT/)CT system. Both MRI- and DEQCT-based methods provide non-invasive means to characterize bone marrow composition and may help improve the accuracy of red bone marrow absorbed dose estimation. If available, the patient-specific red bone marrow distribution could be integrated in the presented workflow.

The observed inter-patient variability in the red bone marrow absorbed dose is primarily related to differences in tumor burden and its impact on our automated segmentation pipeline. As described in the Methods, bone lesion segmentation was performed using an SUV threshold of 2, followed by Otsu thresholding. In patients with high skeletal uptake, the Otsu-derived thresholds are elevated, which may result in minimally or moderately accumulating bone lesions not being classified as lesions. Consequently, these skeletal sites are treated as bone-lesion-free regions, which can lead to an overestimation of the red bone marrow self-absorbed dose. This represents a known limitation of the current workflow, particularly in patients with extensive skeletal metastases. Importantly, these patients were included in the dosimetric analysis to ensure that the workflow was tested in cases with both limited and extensive skeletal metastases. Future improvements may involve more robust segmentation techniques to improve accuracy in patients with high tumor burden.

Deep learning-based CT segmentation tools such as TotalSegmentator [14] are essential for supporting automated dosimetry workflows, offering robust and fast segmentation that would not be feasible manually. Using automated segmentation, image-based bone marrow dosimetry can be based on all skeletal sites visible in the SPECT/CT field of view, rather than relying on a few regions as a surrogate. This represents a major advantage for clinical implementation. Since SPECT/CT imaging typically involves low-dose, non-diagnostic CTs, care must be taken when applying such models in cases of uncommon pathologies or low-contrast regions. Despite this, we observed good segmentation performance in our cohort.

As already stated, the primary objective of this study was to first assess the feasibility of image-based red bone marrow activity quantification in patients with mCRPC treated with [^177^Lu]Lu-PSMA using a large dataset of simulated virtual patient phantoms. Subsequently, we aimed to develop a fully automated dosimetric workflow that enables reproducible red bone marrow absorbed dose estimation without additional manual effort. The presented workflow may help to perform image-based dosimetry for the red bone marrow as an organ at risk during [^177^Lu]Lu-PSMA therapy. Requiring only the reconstructed SPECT images and the corresponding CTs, it may provide automated clinical routine image-based dosimetry with a minimum of required user-interactions. This automated approach is intended to support systematic and large-scale red bone marrow absorbed dose estimation from post-therapeutic imaging, with the long-term goal of supporting hematologic toxicity monitoring and collecting sufficient dosimetric data to ultimately help personalize treatment strategies in [^177^Lu]Lu-PSMA therapy for patients with mCRPC.

## 5. Conclusions

In this work, the feasibility of image-based red bone marrow dosimetry for metastasized prostate cancer was investigated. A simulation study based on 175 virtual phantoms showed that red bone marrow activity estimates can be derived from skeletal sites without lesion infiltration, with uncertainties below 10% in at least 60% of cases. Based on this finding, a workflow for the automated estimation of red bone marrow absorbed doses in clinical practice was presented. Application of the workflow to a cohort of 20 patients, each receiving four cycles, revealed a median total absorbed dose to the red bone marrow of approximately 21 mGy/GBq and demonstrated a trend of decreasing median blood levels with increasing median cumulative total absorbed dose. This workflow may have the potential to support the routine and systematic evaluation of dosimetry data along with clinical response, which is a prerequisite for intensified investigations of the absorbed-dose-response relationships.

## Figures and Tables

**Table 1:** Overview of VOI selection in different skeletal sites.

Method	Skeletal Site	Assumed VOI for the Red Bone Marrow
1	with bone lesions	eroded bone compartment VOI excluding dilated bone lesion VOIs
2	without bone lesions, neighboring	eroded bone compartment VOI
3	without bone lesions, distant	eroded bone compartment VOI

## Data Availability

The data presented in this study can be made available from the corresponding author upon reasonable request.

## Supplementary Materials

The following supporting information can be downloaded at https://www.mdpi.com/article/10.3390/cancers17142313/s1, Figure S1: RCs for the NEMA spheres as a function of $n_{iter}$ for IY using ground truth masks. Figure S2: CNRs for the NEMA spheres as a function of $n_{iter}$ for IY using ground truth masks. Figure S3: RCs for the NEMA spheres as a function of $n_{iter}$ for IY using Otsu-derived masks. Figure S4: CNRs for the NEMA spheres as a function of $n_{iter}$ for IY using Otsu-derived masks. Figure S5: RCs for the NEMA spheres as a function of $n_{iter}$ for LR. Figure S6: CNRs for the NEMA spheres as a function of $n_{iter}$ for LR. Figure S7: Median RCs per virtual phantom for methods 1–3 from Table 1, with and without spill-over reduction, shown for 15 s per projection with Poisson noise applied. Figure S8: Median RCs per virtual phantom for methods 1–3 from Table 1, with and without spill-over reduction, shown for 15 s per projection without Poisson noise. Figure S9: Median RCs per virtual phantom for methods 1–3 from Table 1, depending on time per projection and the presence or absence of Poisson noise, shown for images processed with IY using ground truth masks. Figure S10: Median RCs per virtual phantom for methods 1–3 from Table 1, depending on time per projection and the presence or absence of Poisson noise, shown for images processed with IY using Otsu-derived masks. Figure S11: Median RCs per virtual phantom for methods 1–3 from Table 1, depending on time per projection and the presence or absence of Poisson noise, shown for images processed with LR. Figure S12: Median RCs per virtual phantom for methods 1–3 from Table 1, depending on the selected VOI volume threshold, shown for the default clinical regime (5 s per projection with Poisson noise applied) and images processed with IY using ground truth masks. Figure S13: Median RCs per virtual phantom for methods 1–3 from Table 1, depending on the selected VOI volume threshold, shown for the default clinical regime (5 s per projection with Poisson noise applied) and images processed with IY using Otsu-derived masks. Figure S14: Median RCs per virtual phantom for methods 1–3 from Table 1, depending on the selected VOI volume threshold, shown for the default clinical regime (5 s per projection with Poisson noise applied) and images processed with LR. Figure S15: Median RCs per virtual phantom for methods 1–3 from Table 1, depending on the selected VOI volume threshold, shown for 15 s per projection with Poisson noise applied and the original reconstructed images. Figure S16: Median RCs per virtual phantom for methods 1–3 from Table 1, depending on the selected VOI volume threshold, shown for 15 s per projection with Poisson noise applied and images processed with IY using ground truth masks. Figure S17: Median RCs per virtual phantom for methods 1–3 from Table 1, depending on the selected VOI volume threshold, shown for 15 s per projection with Poisson noise applied and images processed with IY using Otsu-derived masks. Figure S18: Median RCs per virtual phantom for methods 1–3 from Table 1, depending on the selected VOI volume threshold, shown for 15 s per projection with Poisson noise applied and images processed with LR. Figure S19: Median RCs per virtual phantom for methods 1–3 from Table 1, depending on the selected VOI volume threshold, shown for 15 s per projection without Poisson noise and the original reconstructed images. Figure S20: Median RCs per virtual phantom for methods 1–3 from Table 1, depending on the selected VOI volume threshold, shown for 15 s per projection without Poisson noise and images processed with IY using ground truth masks. Figure S21: Median RCs per virtual phantom for methods 1–3 from Table 1, depending on the selected VOI volume threshold, shown for 15 s per projection without Poisson noise and images processed with IY using Otsu-derived masks. Figure S22: Median RCs per virtual phantom for methods 1–3 from Table 1, depending on the selected VOI volume threshold, shown for 15 s per projection without Poisson noise and images processed with LR. Figure S23: RCs estimated in the kidneys and bone lesions for all virtual patient phantoms. The minimum analyzed bone lesion volume is 1 mL. Figure S24: First-cycle SPECT images at 24 h p.i. for patients with patient IDs 2, 5, 9 and 14 (from left to right). Figure S25: First-cycle SPECT images at 24 h p.i. for patients with patient IDs 1, 3, 4 and 6 (from left to right). Figure S26: Blood levels of erythrocytes, lymphocytes, and thrombocytes prior to each injection across four therapy cycles. Units: G/L = $10^{9}$ cells per liter; T/L = $10^{12}$ cells per liter. Paired Wilcoxon signed-rank tests with a one-sided hypothesis (“greater”) were performed to assess whether blood parameter values at earlier cycles were significantly higher than those at later cycles. p-values are annotated accordingly. Table S1: Estimated median RCs across all virtual phantoms for methods 1–3 from Table 1, including the influence of spill-over reduction, time per projection, Poisson noise and VOI volume threshold. Values for the default clinical regime are shown in bold. References [7,14,15,16,30] are cited in the Supplementary Materials.

## Abbreviations

The following abbreviations are used in this manuscript:

CNR	Contrast-to-Noise Ratio
CT	Computed Tomography
DEQCT	Dual-Energy Quantitative Computed Tomography
EANM	European Association of Nuclear Medicine
FWHM	Full Width at Half Maximum
HU	Hounsfield Units
ICRP	International Commission on Radiological Protection
IY	Iterative Yang
LR	Lucy–Richardson
mCRPC	Metastatic Castration-Resistant Prostate Cancer
mHSPC	Metastatic Hormone-Sensitive Prostate Cancer
MRI	Magnetic Resonance Imaging
NEMA	National Electrical Manufacturers Association
NET	Neuroendocrine Tumors
OLINDA/EXM	Organ Level Internal Dose Assessment/Exponential Modeling
p.i.	Post-Injection
PET	Positron Emission Tomography
PSF	Point Spread Function
PSMA	Prostate-Specific Membrane Antigen
PVC	Partial Volume Correction
RC	Recovery Coefficient
SPECT	Single Photon Emission Computed Tomography
SUV	Standardized Uptake Value
TAC	Time-Activity Curve
TIA	Time-Integrated Activity
VOI	Volume of Interest
VSV	Voxel-S-Value
