# Supplementary material for: Feasibility of Automated Image-Based Red Bone Marrow Dosimetry for [177Lu]Lu-PSMA Radiopharmaceutical Therapy of Metastatic Castration-Resistant Prostate Cancer
Source: Cancers (Basel). 2025 Jul 11;17(14):2313. doi: 10.3390/cancers17142313 (PMC12294081; doi:10.3390/cancers17142313)
Supplement: Supplementary file 1 [file cancers-17-02313-s001.zip › cancers-3685039-supplementary.pdf]

Supplementary information

Simulation Data

To define virtual patient phantoms with a realistic distribution of bone metastases, 639 pre-therapeutic [^{18}F]F-PSMA-1007 PET/CT scans were analyzed. First, all CT images were segmented using TotalSegmentator [1]. CTs (shape: 512 x 512 x N with N>200; voxels: 0.986 mm x 0.98 mm x 3 mm) and CT-based segmentations were resampled to match the corresponding PET images (shape: 200 x 200 x M with M>200; voxels: 4.07 mm x 4.07 mm x 2 mm). Subsequently, Otsu thresholding [2] was applied to the PET signal in the CT-based kidney segmentation to define binary masks for the renal cortices. For the creation of bone lesion masks, a standardized uptake value (SUV) threshold of 2 was applied to all bone segments prior to Otsu thresholding to remove non-specific uptake in the bones. Bone lesions with an individual volume of less than 1 mL were excluded.

In the resulting dataset of 639 segmented PET scans, the final database of virtual phantoms was derived by excluding patients with a segmented total bone lesion volume of less than 10 mL, which resulted in 175 virtual patient phantoms. Density maps, as required for simulating SPECT projection data using SIMIND [3], were defined by converting the Hounsfield values HU in the downsampled CT data to densities ρ using a camera-specific bi-linear calibration. To define the activity maps for SIMIND, first, the activity concentrations (mean and standard deviation) in the bone lesions, kidneys and background compartment were extracted from real SPECT images at 24 h post-injection (p.i.), acquired after the first treatment of five patients with 7.4 GBq [^{177}Lu]Lu-PSMA-I&T (mean ratio bone lesions:kidneys:background 40:20:1). For the virtual phantom case, the assumed activity concentration per compartment was then uniformly sampled from the interval mean +/- standard deviation.

Simulation

SIMIND simulations were performed on the Linux Cluster of the Leibniz Supercomputing Centre of the Bavarian Academy of Sciences and Humanities (Garching/Munich, Germany). The simulation parameters were selected to mimic a clinical SPECT acquisition of patients undergoing [^{177}Lu]Lu-PSMA therapy at our department. A Siemens Symbia SPECT scanner (3/8” crystal thickness) mounted with a medium-energy collimator was simulated. The energy windows for the 208 keV gamma emission peak of ^{177}Lu were: main photopeak at 208 keV (width 15 %); lower scatter window at 170 keV (width: 15%); upper scatter window at 240 keV (width: 10%). SPECT raw data was further acquired in 128x128 pixels (4.7952 mm x 4.7952 mm) over 128 angles. The number of simulated bed positions was set to three to capture the primary activity-accumulating region of the body, spanning from below the eyes to approximately the middle of the thighs. The total number of simulated photons was set to 10 billion to generate low-noise projection data.

Reconstruction

The simulated high-count SPECT projections (unit: counts/(MBq*s)) were scaled with the total activity of each virtual patient and the clinical acquisition time per projection of 5 s. Poisson noise was applied to the scaled projection data. An in-house MAP-OSEM algorithm (Bayesian weight 0.001) with attenuation correction, triple-energy-window scatter correction and Gaussian resolution modelling enabled was used to reconstruct the SPECT images based on the simulated SPECT projection data. The counts in the reconstructed SPECT images were converted to kBq/mL using a calibration factor, pre-determined based on a simulation of a cylinder containing a homogeneous activity distribution. The final dataset comprised 175 pairs of reference and reconstructed virtual activity distributions.

Optimal number of iterations for IY and LR

Figures S1–S6 show the RCs and CNRs for the NEMA spheres as a function of the number of iterations for IY with the ground truth and Otsu masks and for LR.

Comparison of original reconstruction and spill-over reduction techniques

Figures S7 and S8 show the results for the datasets with i) time per projection set to 15s instead of 5s and applying Poisson noise and ii) time per projection set to 15s without application of Poisson noise.

Effect of time per projection and Poisson noise on RC estimation

Figures S9-S11 show the results for the images after applying the spill-over reduction techniques.

Effect of VOI volume threshold on RC estimation

Figures S12-S22 show the results for the original and corrected reconstructed images across different acquisition settings.

In Figures S7-S11, the minimum volume of analyzed skeletal VOIs is 1 mL. In Figures S7-S22, the green band indicates the RC range of 90–110%, provided for visual orientation; vertical fractions show the number of virtual phantoms (out of 175) with median RCs within the green band.

RCs in the kidneys and bone lesions

When applying spill-over reduction techniques such as IY or LR, it is also important to check the RCs in the kidneys and bone lesions. Figure S23 shows the estimated RCs in the kidneys and bone lesions for all virtual patient phantoms before and after applying spill-over reduction techniques. As

expected, the application of both IY or LR to the original reconstructed images resulted in increased median RCs for both the kidneys and bone lesions. Similar to the RCs for skeletal VOIs, for the bone lesions, IY with the ground truth bone lesion masks outperformed IY with the Otsu bone lesion masks, which yielded a better median RC compared to LR deconvolution. Interestingly, for the kidneys, LR delivered a slightly higher median RC than IY with the Otsu kidney masks.

Real patient data

Figures S24 and S25 show the first-cycle SPECT images at 24 h p.i. for patients with high and low total tumor volume.

Estimation of the cross-absorbed dose to the red bone marrow

The cross-absorbed dose to the red bone marrow D_{rbm}^{cross} was estimated according to the EANM guideline [4]:

$$D_{RBM}^{cross} = \sum_{h} \tilde{A}_h S_{RBM \leftarrow h} + \tilde{A}_{RoB} S_{RBM \leftarrow RoB}$$

$$= (\tilde{A}_{LK} + \tilde{A}_{RK}) S_{RBM \leftarrow K} + (\tilde{A}_{TB} - \tilde{A}_{RBM} - \tilde{A}_{LK} - \tilde{A}_{RK}) S_{RBM \leftarrow RoB},$$

where $\sum_h$ ⬚ means summation over organs 'h' other than total body and the red bone marrow itself contributing to the cross-absorbed dose to the red bone marrow (assumed to be the left and the right kidney); $\tilde{A}$ is the number of decays in the respective region; 'RBM', 'RoB', 'LK', 'RK', 'K', 'TB' represent the red bone marrow, remainder of the body, left kidney, right kidney, both kidneys and total body, respectively; $S_{target \leftarrow source}$ is the S-value of a source leading to an absorbed dose in the target. The patient S-values are calculated based on the phantom S-values from OLINDA/EXM [5] as follows:

$$S_{RBM \leftarrow RoB,patient}$$

$$= S_{RBM \leftarrow TB,phantom} \frac{m_{TB,pahntom}}{m_{RoB,patient}} \frac{m_{RBM,phantom}}{m_{RBM,patient}}$$

$$- \ S_{RBM \leftarrow RBM,phantom} \frac{m_{RBM,phantom}}{m_{RoB,patient}} \frac{m_{RBM,phantom}}{m_{RBM,patient}}$$

$$- S_{RBM \leftarrow K,phantom} \frac{m_{K,phantom}}{m_{RoB,patient}} \frac{m_{RBM,phantom}}{m_{RBM,patient}}$$

$$\approx \frac{m_{TB,phantom}^{2}}{m_{RoB,phantom} m_{TB,patient}^{2}} \left[S_{RBM \leftarrow TB,phantom} m_{TB,phantom} \right.$$

$$\left. - S_{RBM \leftarrow RBM,phantom} m_{RM,phantom} - S_{RBM \leftarrow K,phantom} m_{K,phantom} \right]$$

$$S_{RBM \leftarrow K,patient} = S_{RBM \leftarrow K,phantom} \frac{m_{K,phantom}}{m_{K,patient}} \frac{m_{RBM,phantom}}{m_{RBM,patient}}$$

$$\approx S_{RBM \leftarrow K,phantom} \frac{m_{K,phantom}}{m_{K,patient}} \frac{m_{TB,phantom}}{m_{TB,patient}}$$

assuming $\frac{m_{RBM,phantom}}{m_{RBM,patient}} \approx \frac{m_{TB,phantom}}{m_{TB,patient}}$ and $\frac{m_{ROB,phantom}}{m_{RoB,patient}} \approx \frac{m_{TB,phantom}}{m_{TB,patient}}$. In our workflow, $\tilde{A}_{RBM}$ was estimated as follows:

$$\tilde{A}_{RBM} \approx \frac{m_{RBM,patient}}{\sum_k m_{RBM,k,patient}} \sum_i \tilde{A}_{RBM,i} \approx \frac{m_{RBM,phantom}}{\sum_k m_{RBM,k,patient}} \frac{m_{TB,patient}}{m_{TB,phantom}} \sum_i \tilde{A}_{RBM,i}$$

where $m_{RBM,k,patient}$ and $\tilde{A}_{RBM,i}$ represent the mass of the red bone marrow and the number of decays in the red bone marrow in the respective skeletal site, respectively.

The following values from [5] were used:

$$m_{RBM,phantom} = 1170\ g$$

$$m_{TB,phantom} = 73000\ g$$

$$m_{K,phantom} = 310\ g$$

$$m_{RoB,phantom} = m_{TB,phantom} - m_{RBM,phantom} - m_{K,phantom} = \ \ 71520\ g$$

$$S_{RBM \leftarrow TB,phantom} = 2.74 \times 10^{-7} \ \frac{mGy}{MBq \cdot s}$$

$$S_{RBM \leftarrow K,phantom} = 3.35 \times 10^{-8} \frac{mGy}{MBq \cdot s}$$

$$S_{RBM \leftarrow RBM,phantom} = 1.15 \times 10^{-5} \frac{mGy}{MBq \cdot s}$$

The uncertainty of the cross-absorbed dose to the red bone marrow was estimated based on the uncertainties of the TIAs for the total body, left kidney, right kidney, and red bone marrow using propagation of uncertainty.

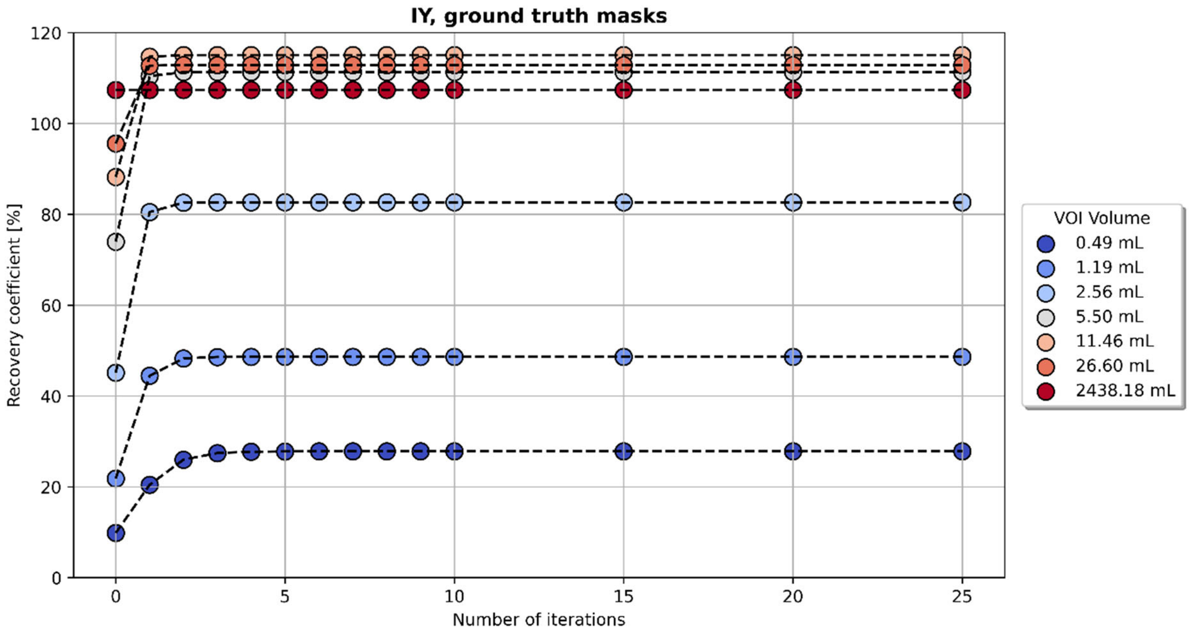

Figure S1. RCs for the NEMA spheres as a function of n_{iter} for IY using ground truth masks.

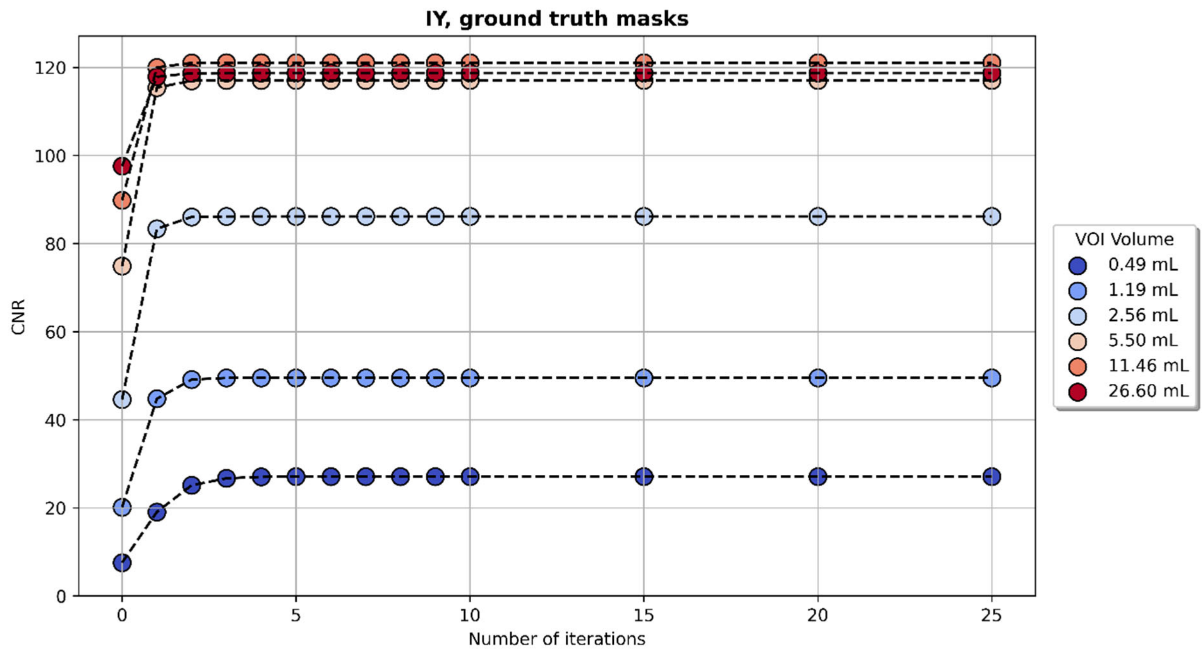

Figure S2. CNRs for the NEMA spheres as a function of n_{iter} for IY using ground truth masks.

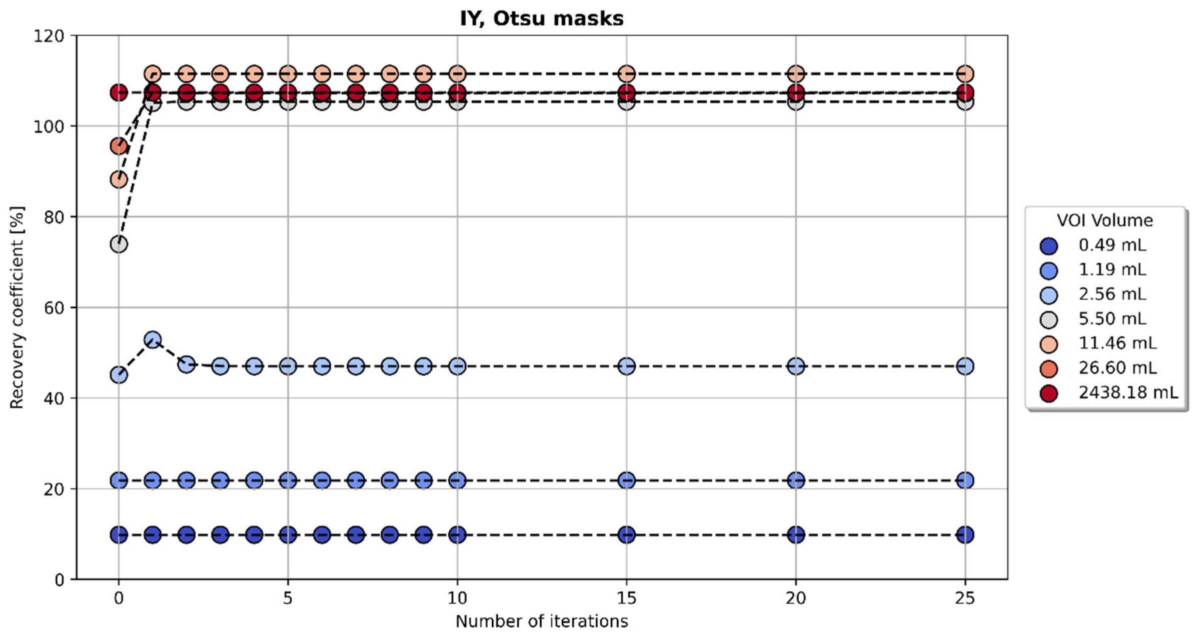

Figure S3. RCs for the NEMA spheres as a function of n_{iter} for IY using Otsu-derived masks.

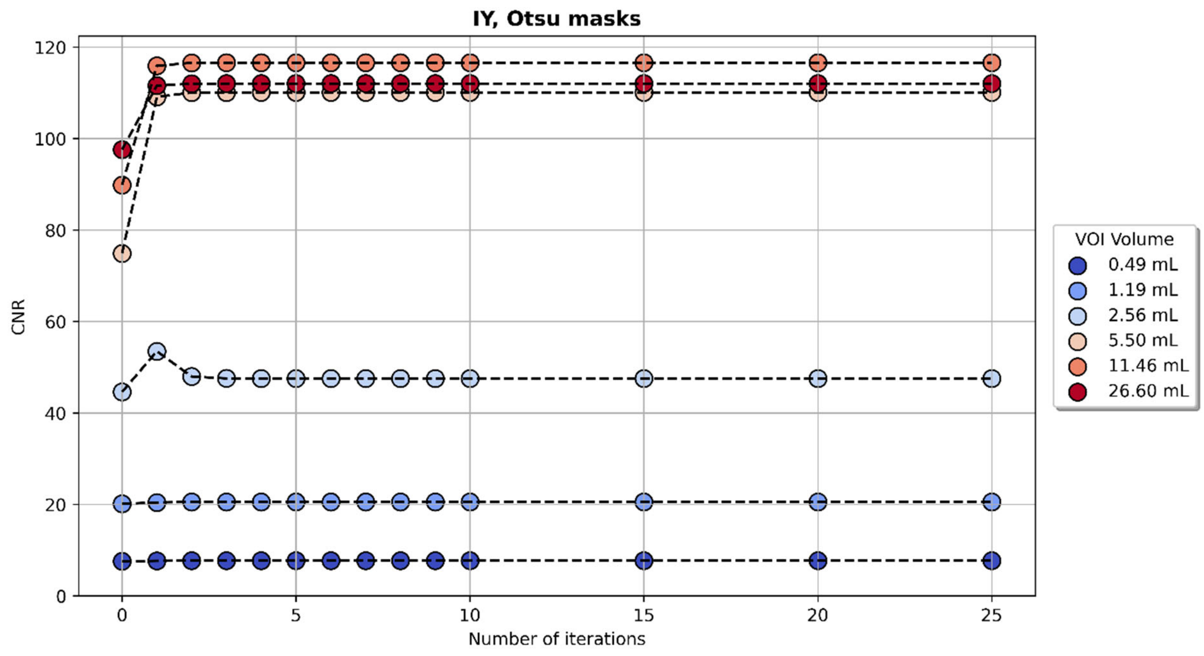

Figure S4. CNRs for the NEMA spheres as a function of n_{iter} for IY using Otsu-derived masks.

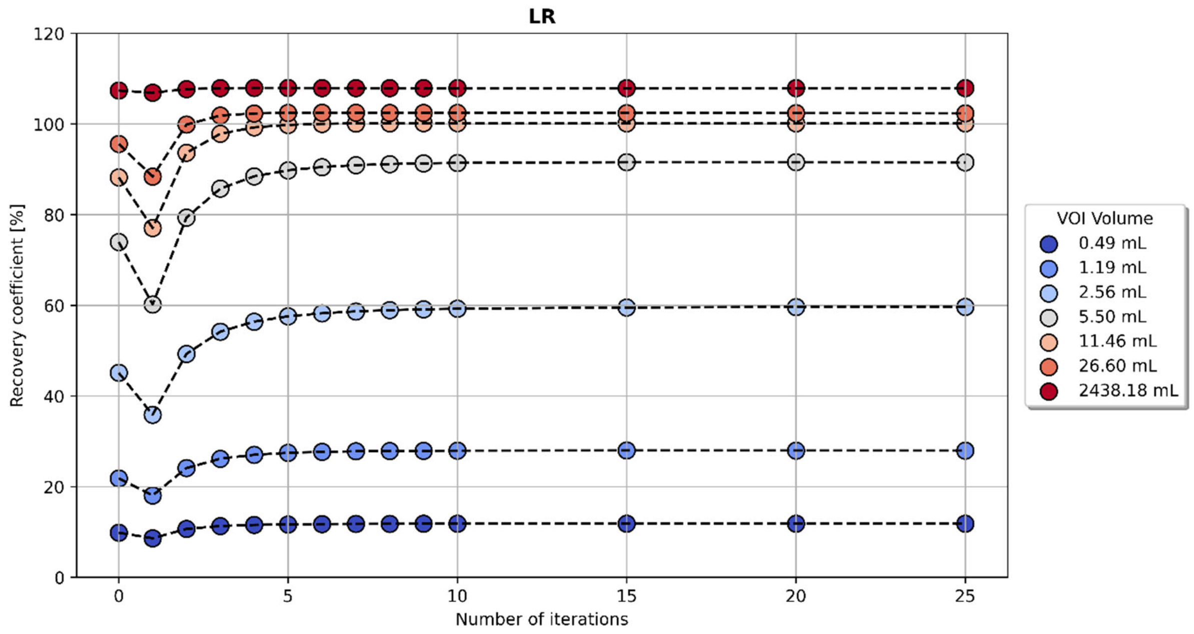

Figure S5. RCs for the NEMA spheres as a function of n_{iter} for LR.

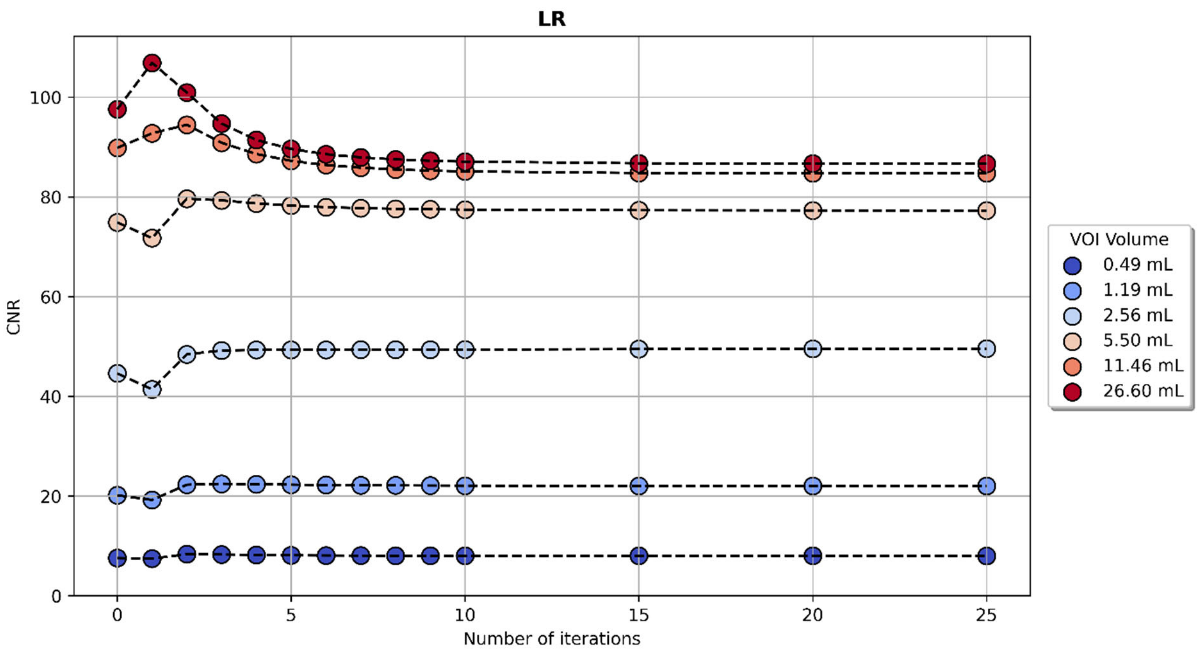

Figure S6. CNRs for the NEMA spheres as a function of n_{iter} for LR.

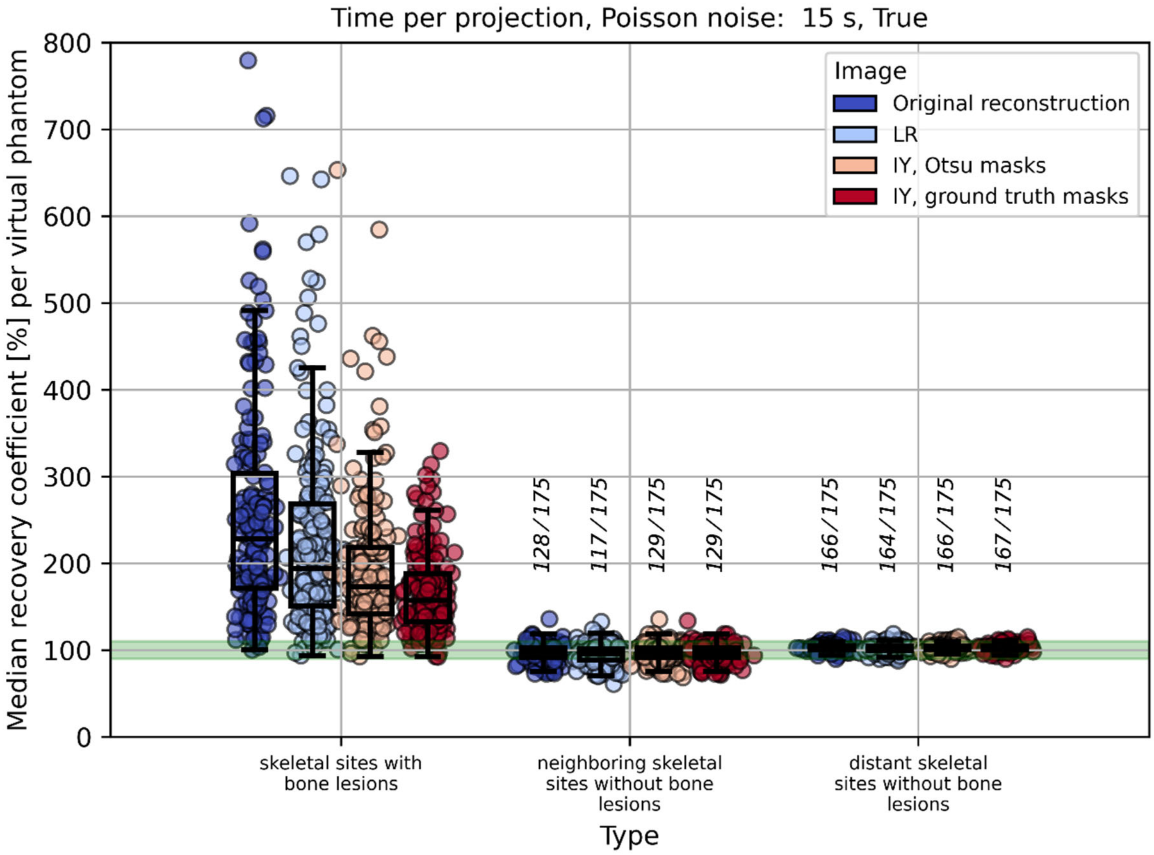

Figure S7. Median RCs per virtual phantom for methods 1-3 from Table 1, with and without spill-over reduction, shown for 15 s per projection with Poisson noise applied.

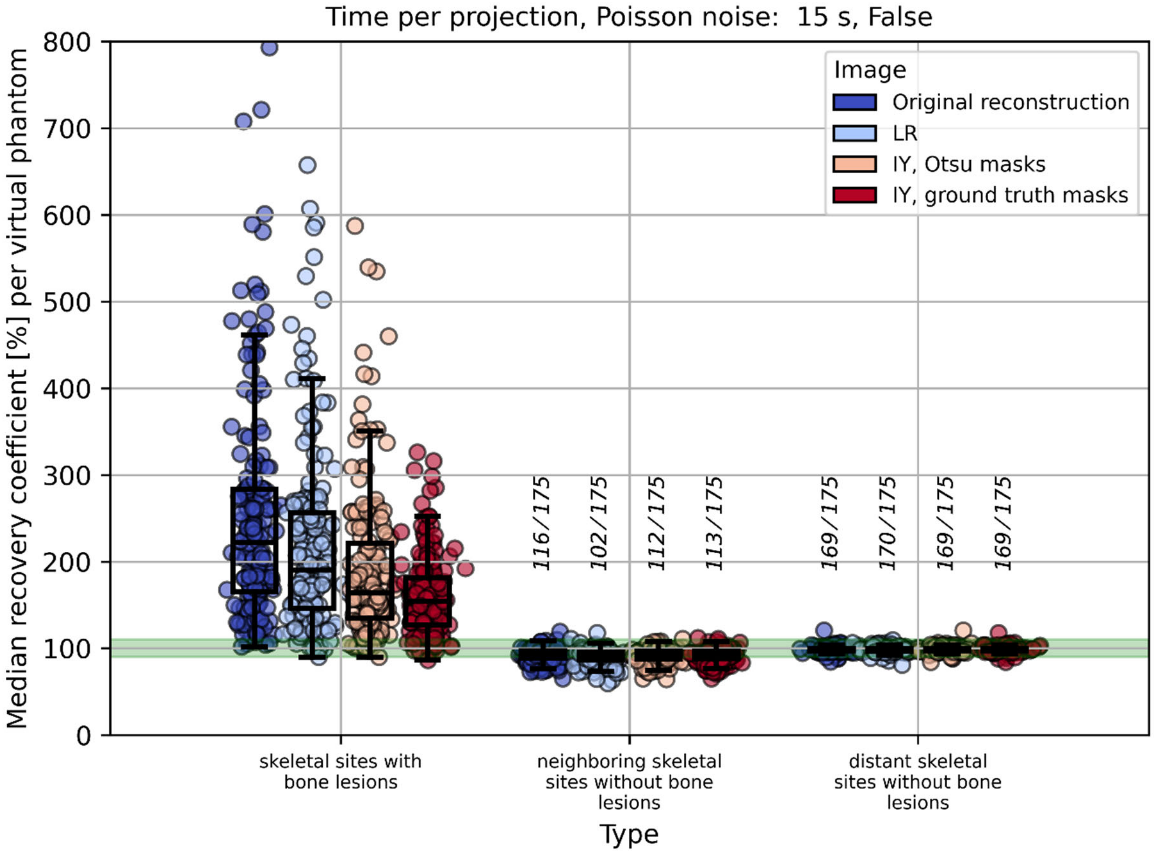

Figure S8. Median RCs per virtual phantom for methods 1-3 from Table 1, with and without spill-over reduction, shown for 15 s per projection without Poisson noise.

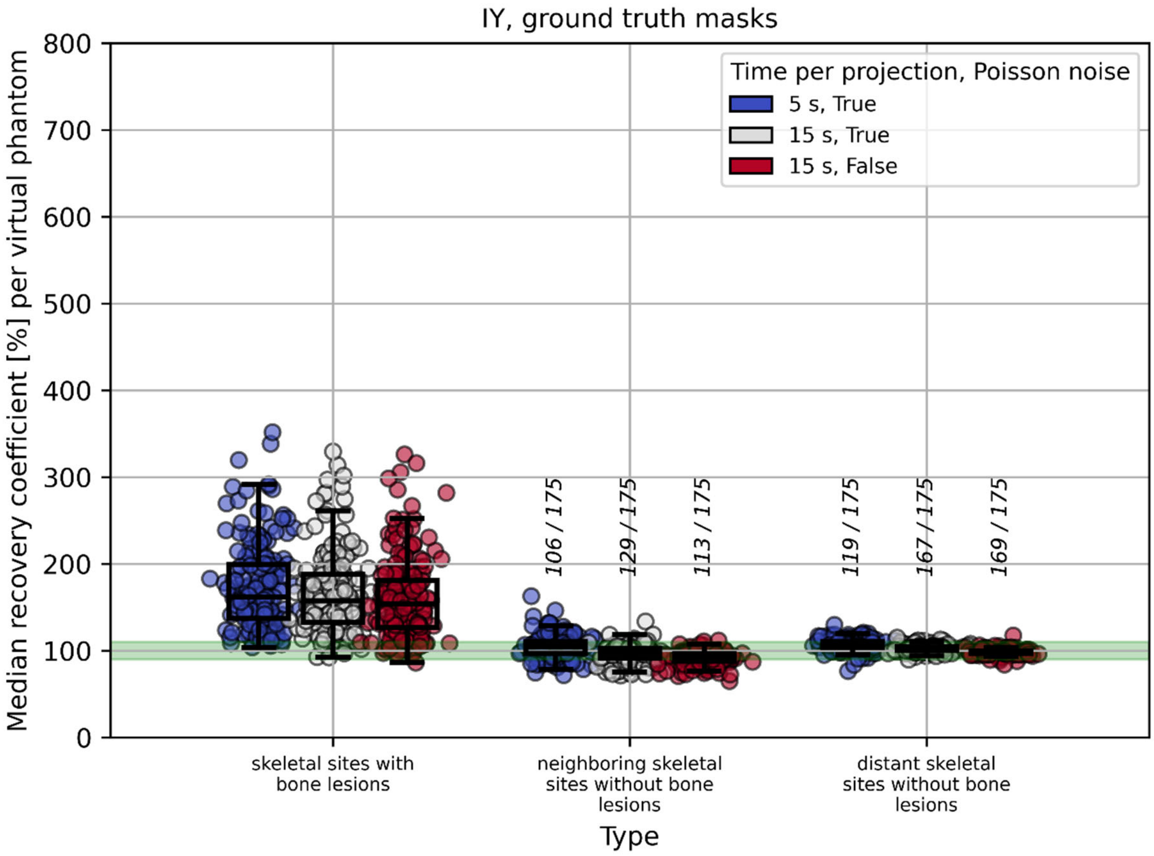

Figure S9. Median RCs per virtual phantom for methods 1-3 from Table 1, depending on time per projection and the presence or absence of Poisson noise, shown for images processed with IY using ground truth masks.

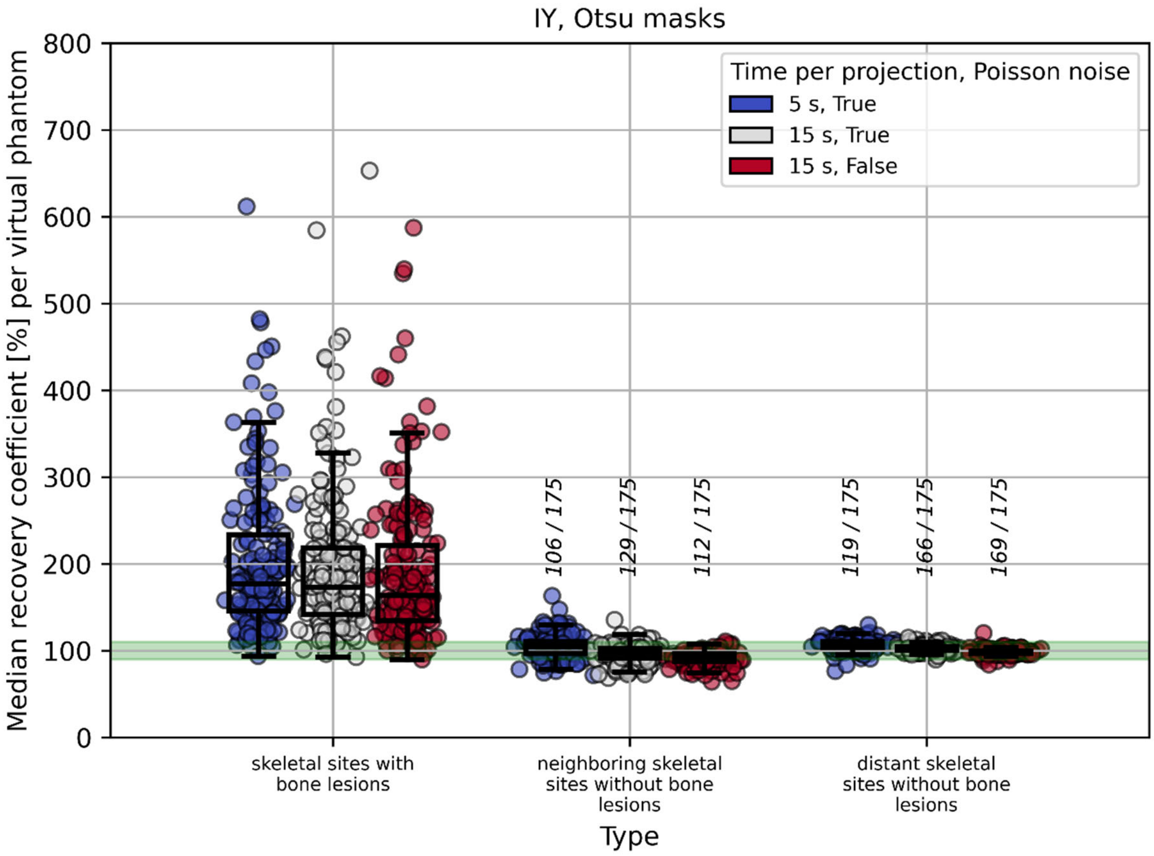

Figure S10. Median RCs per virtual phantom for methods 1-3 from Table 1, depending on time per projection and the presence or absence of Poisson noise, shown for images processed with IY using Otsu-derived masks.

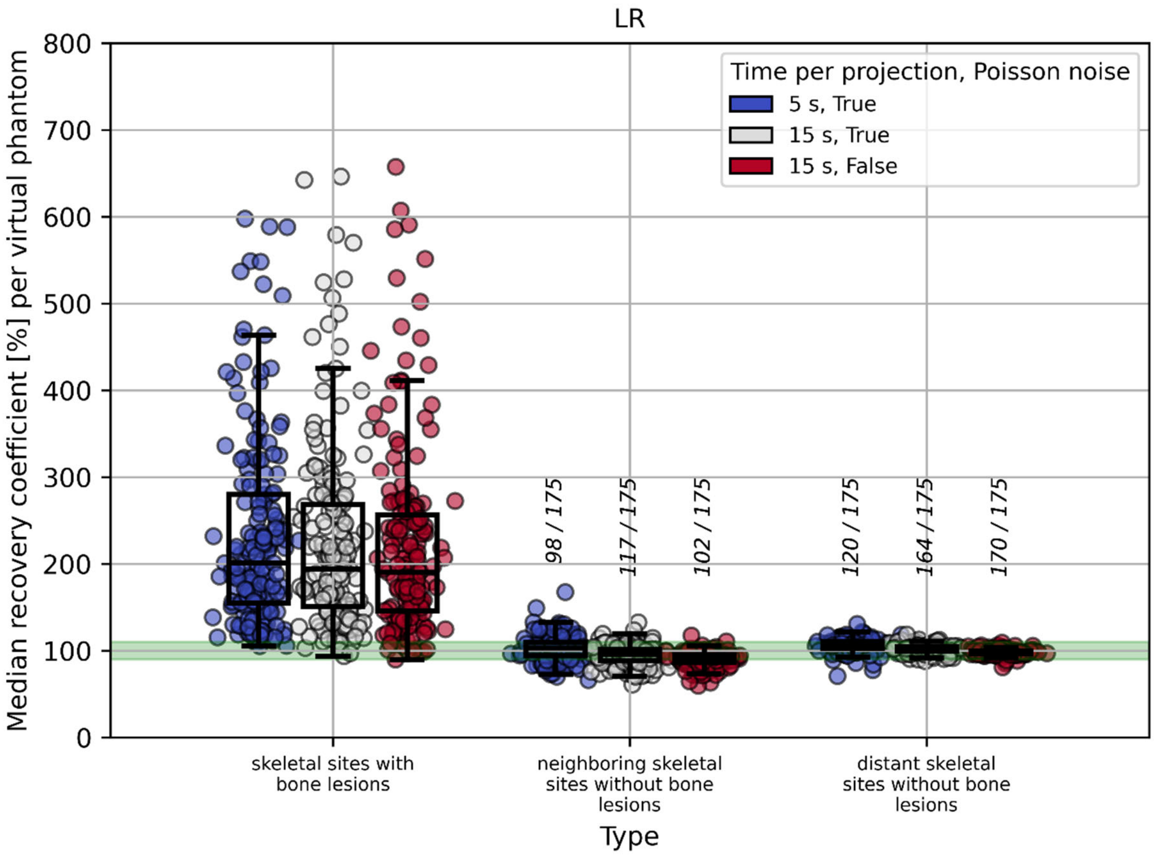

Figure S11. Median RCs per virtual phantom for methods 1-3 from Table 1, depending on time per projection and the presence or absence of Poisson noise, shown for images processed with LR.

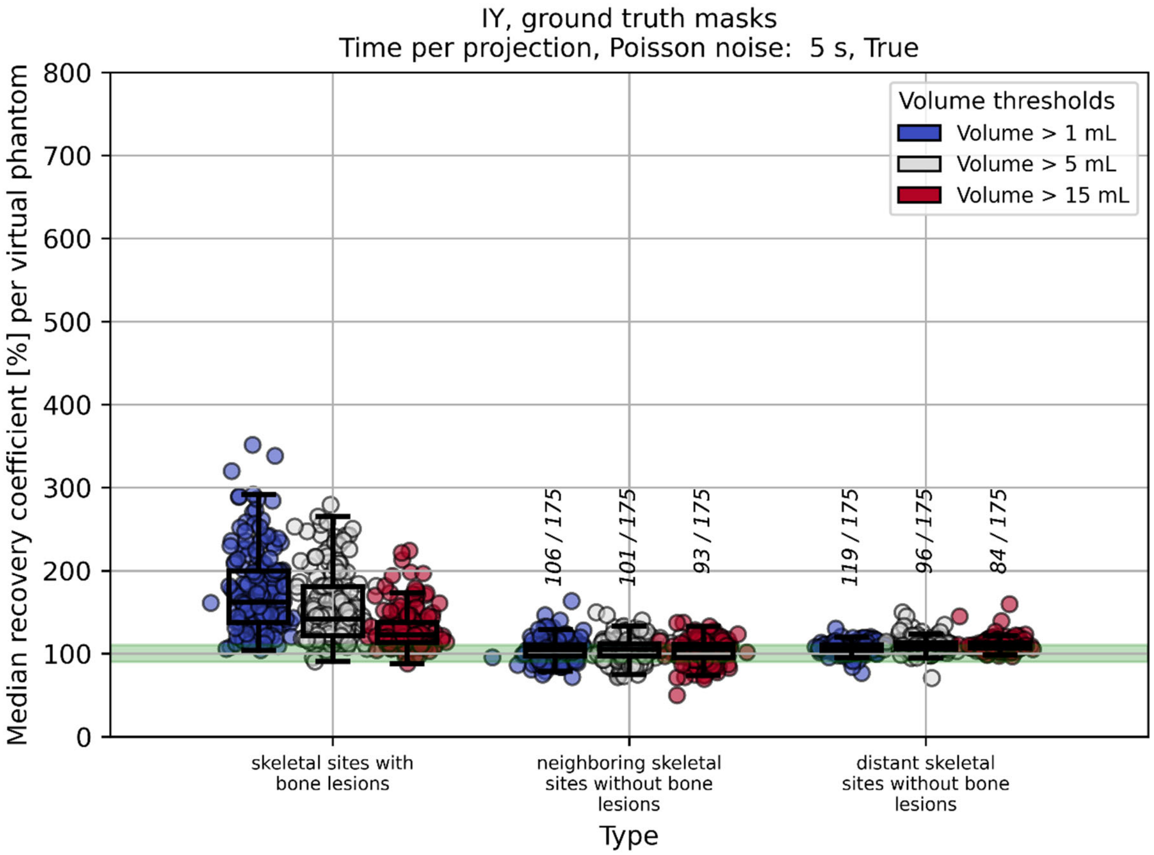

Figure S12. Median RCs per virtual phantom for methods 1-3 from Table 1, depending on the selected VOI volume threshold, shown for the default clinical regime (5 s per projection with Poisson noise applied) and images processed with IY using ground truth masks.

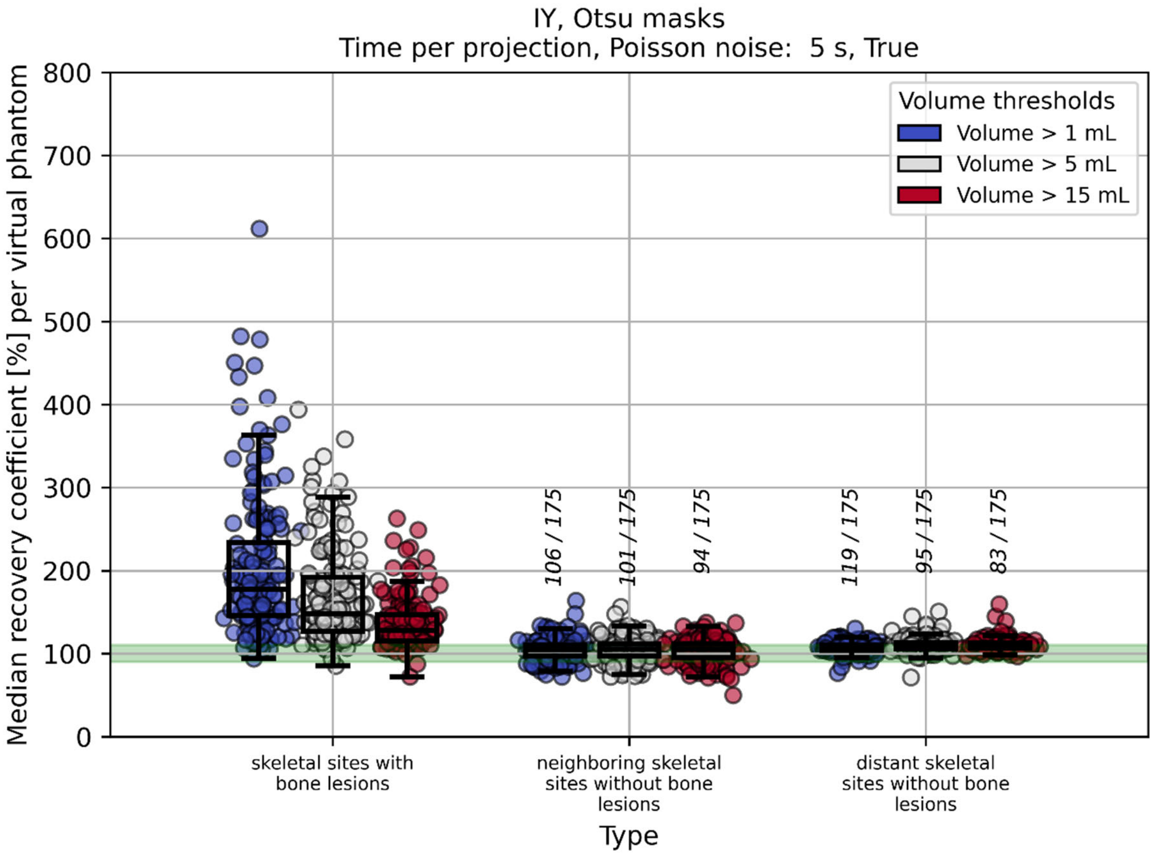

Figure S13. Median RCs per virtual phantom for methods 1-3 from Table 1, depending on the selected VOI volume threshold, shown for the default clinical regime (5 s per projection with Poisson noise applied) and images processed with IY using Otsu-derived masks.

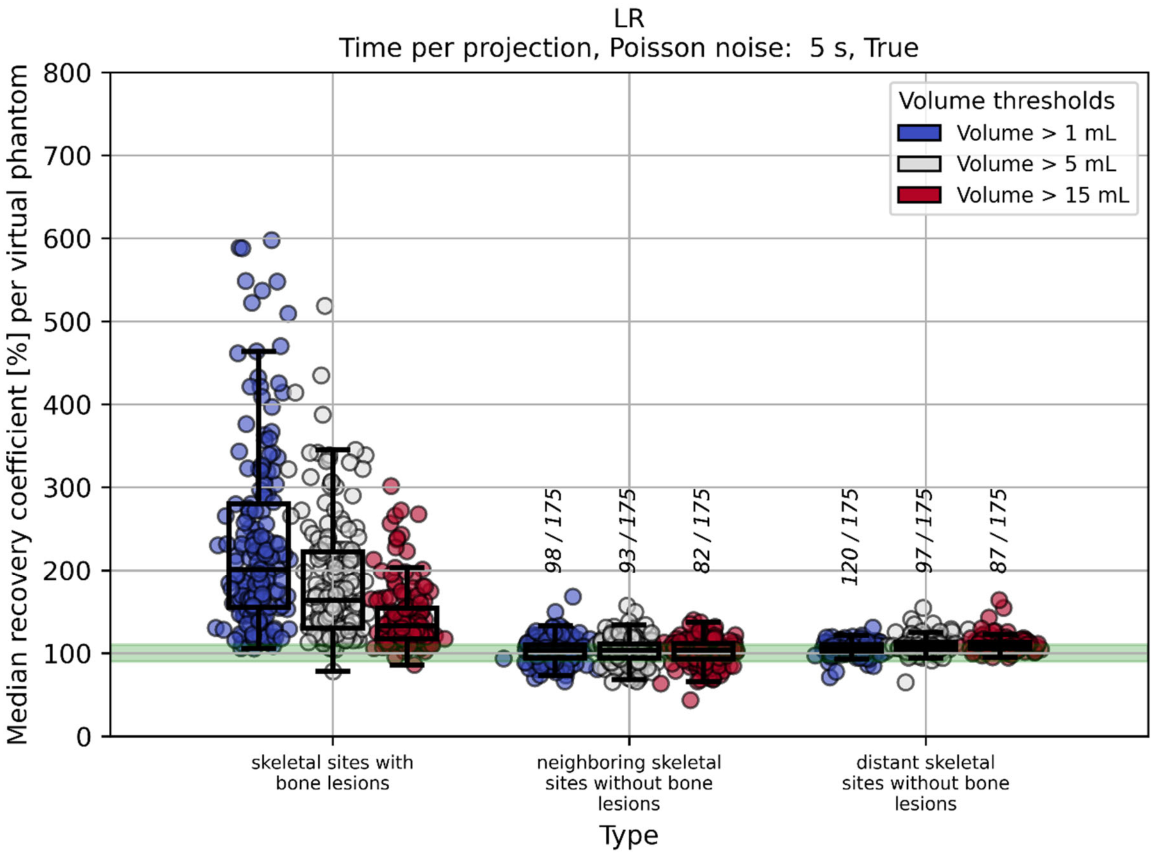

Figure S14. Median RCs per virtual phantom for methods 1-3 from Table 1, depending on the selected VOI volume threshold, shown for the default clinical regime (5 s per projection with Poisson noise applied) and images processed with LR.

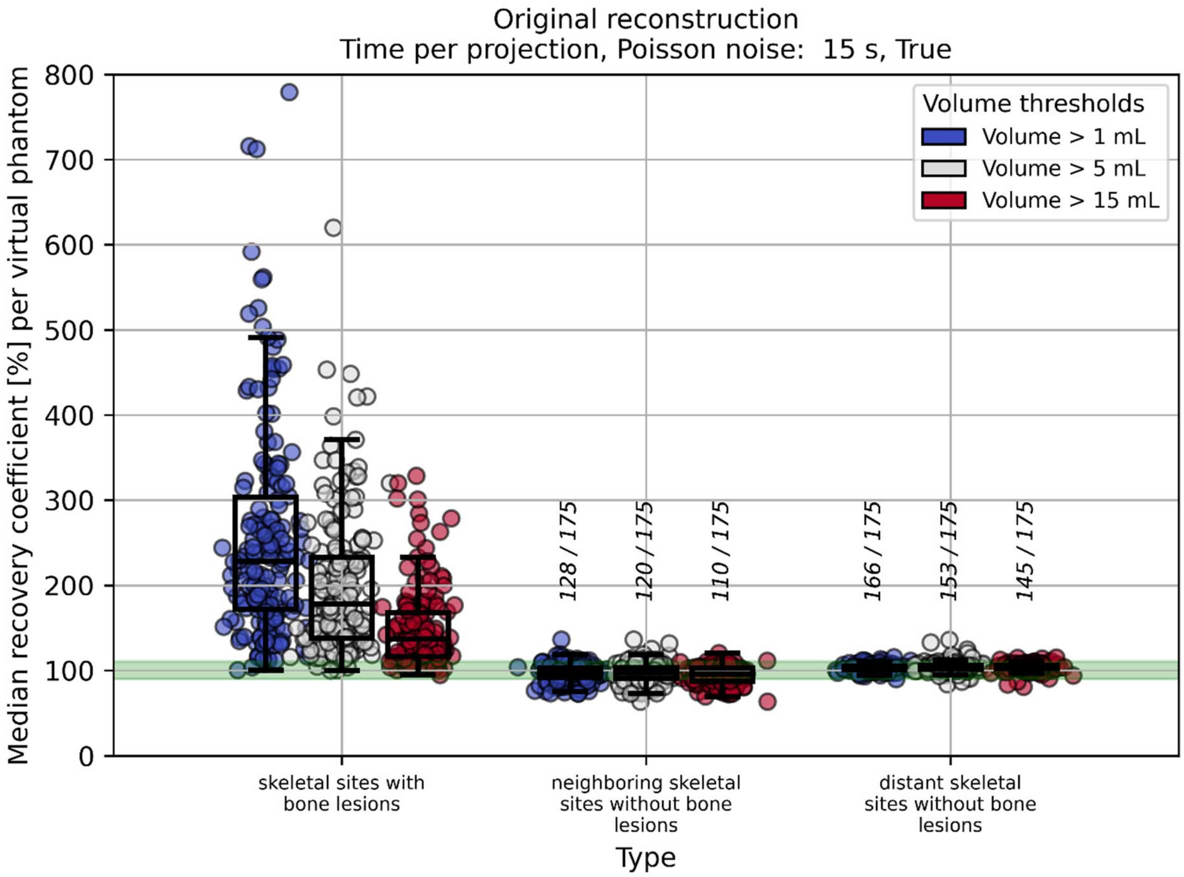

Figure S15. Median RCs per virtual phantom for methods 1-3 from Table 1, depending on the selected VOI volume threshold, shown for 15 s per projection with Poisson noise applied and the original reconstructed images.

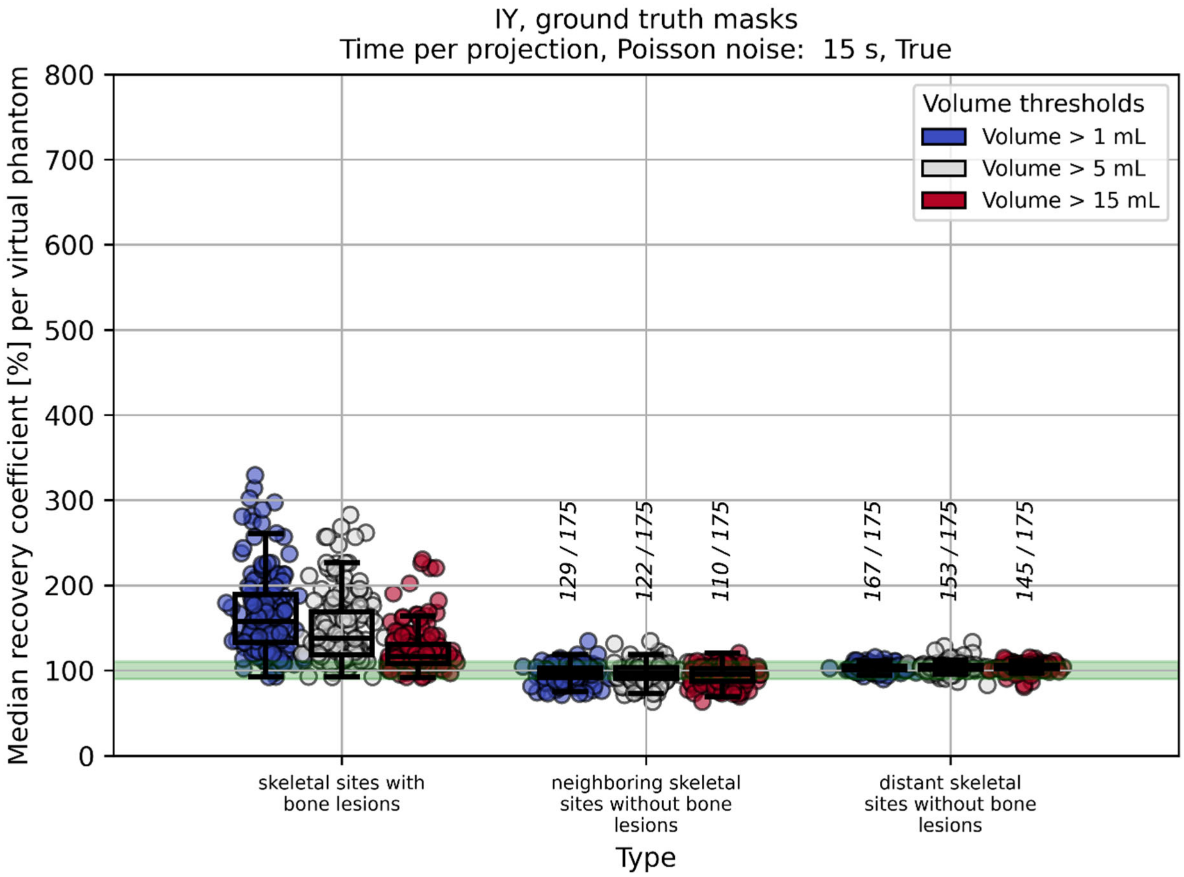

Figure S16. Median RCs per virtual phantom for methods 1-3 from Table 1, depending on the selected VOI volume threshold, shown for 15 s per projection with Poisson noise applied and images processed with IY using ground truth masks.

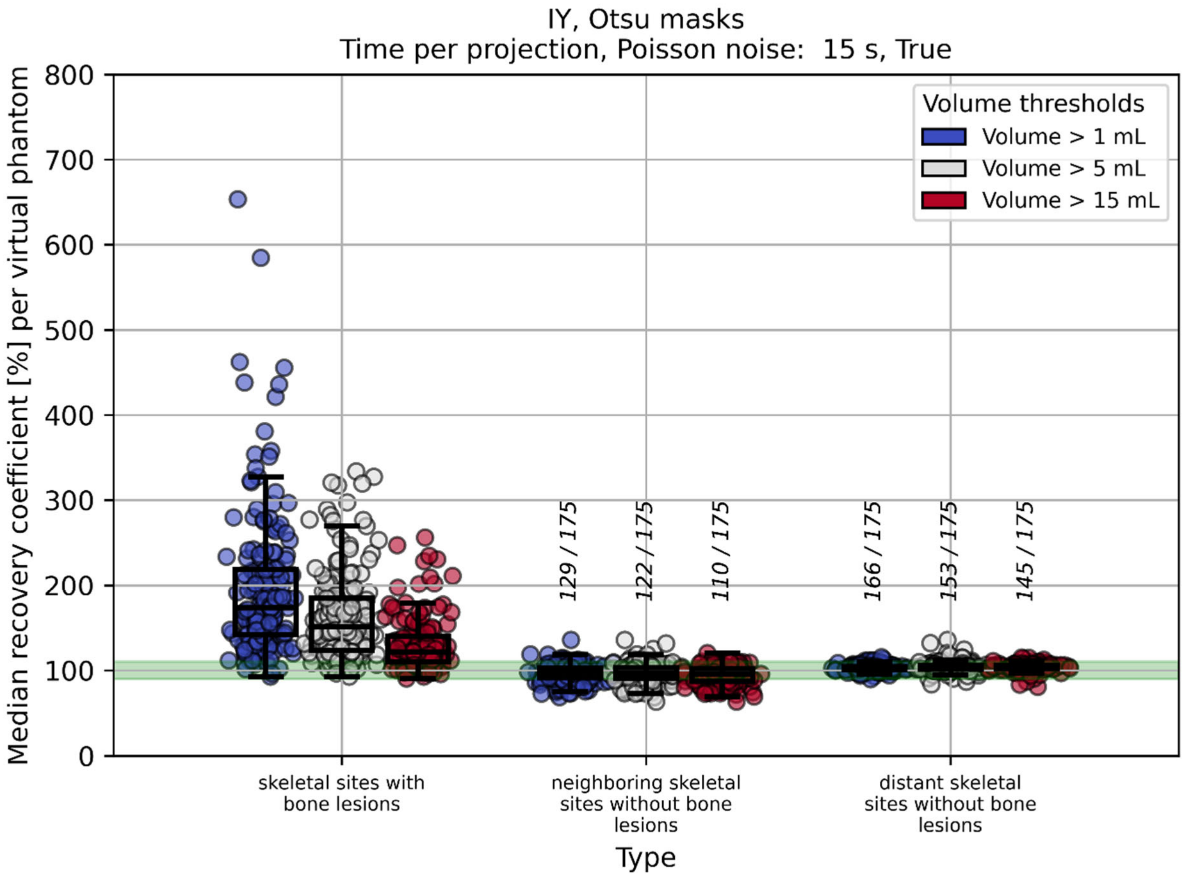

Figure S17. Median RCs per virtual phantom for methods 1-3 from Table 1, depending on the selected VOI volume threshold, shown for 15 s per projection with Poisson noise applied and images processed with IY using Otsu-derived masks.

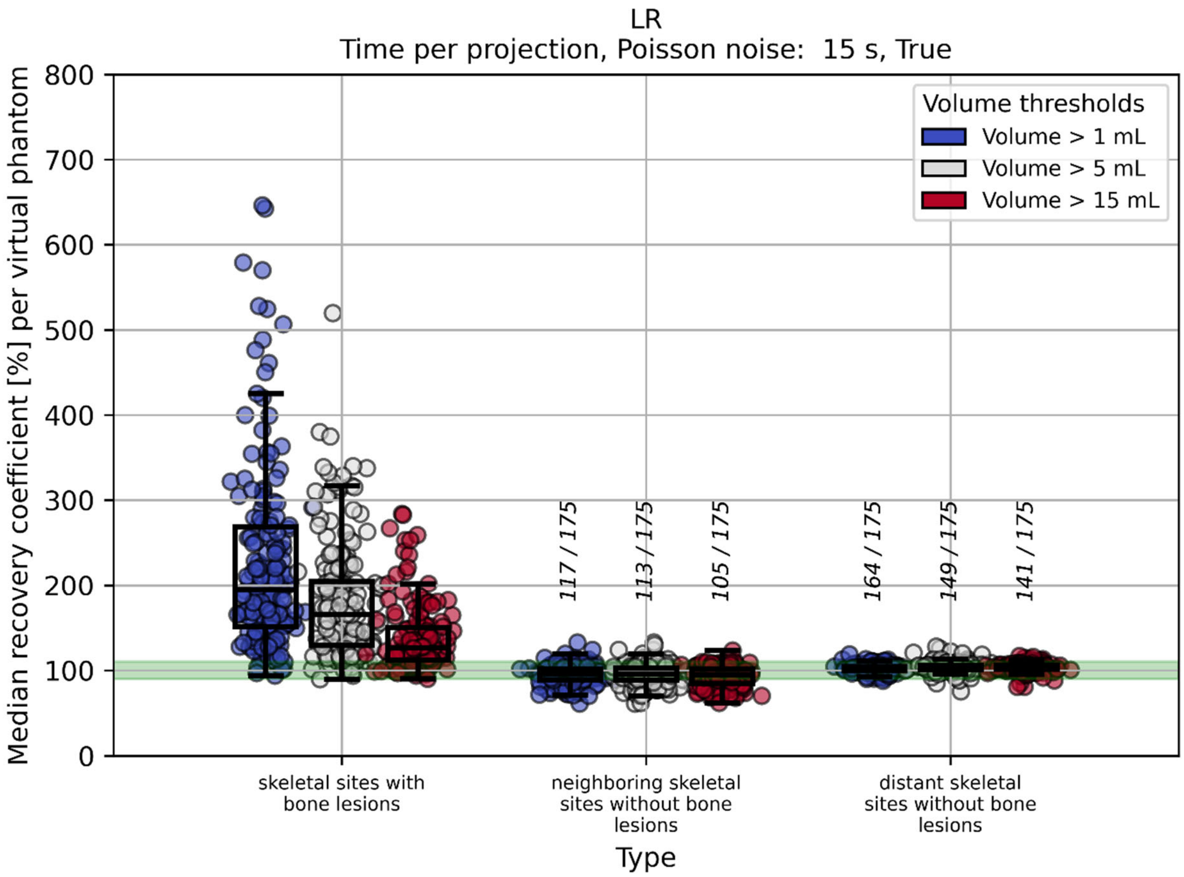

Figure S18. Median RCs per virtual phantom for methods 1-3 from Table 1, depending on the selected VOI volume threshold, shown for 15 s per projection with Poisson noise applied and images processed with LR.

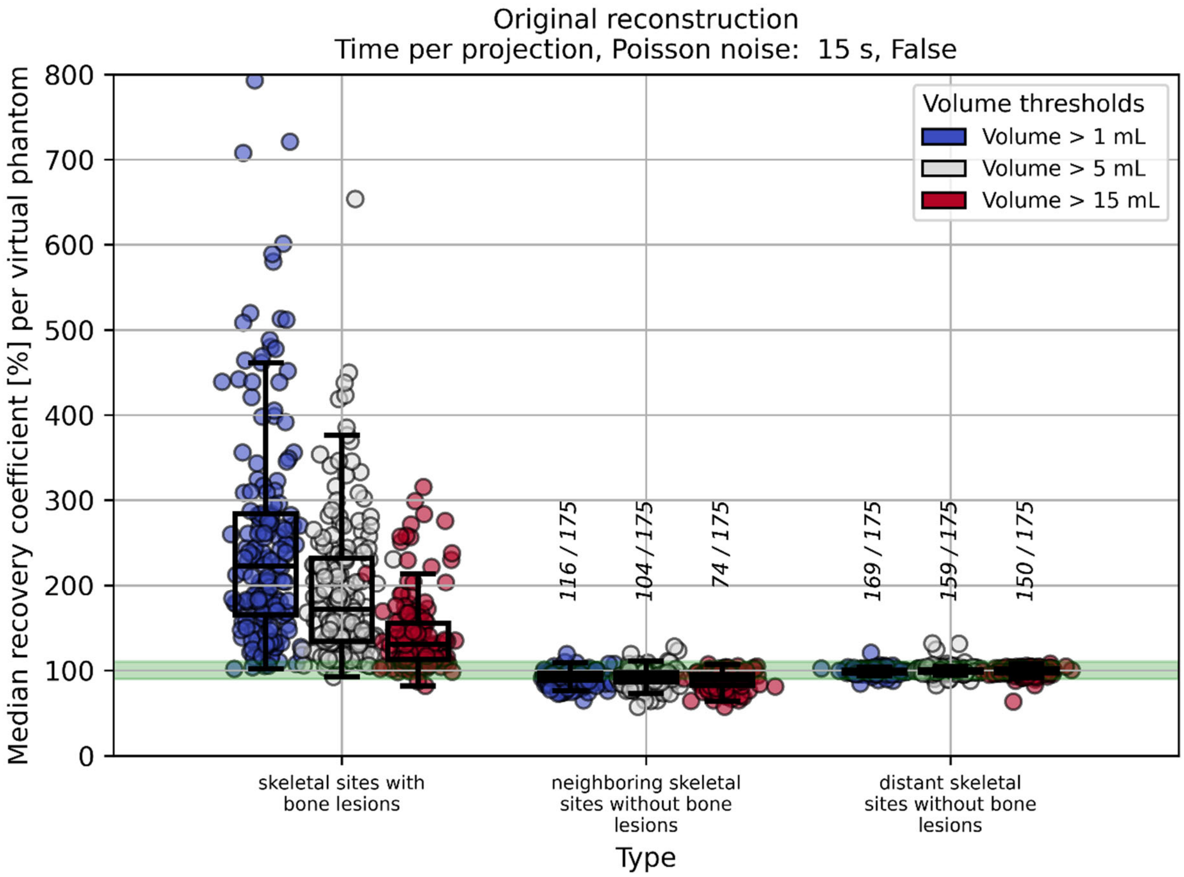

Figure S19. Median RCs per virtual phantom for methods 1-3 from Table 1, depending on the selected VOI volume threshold, shown for 15 s per projection without Poisson noise and the original reconstructed images.

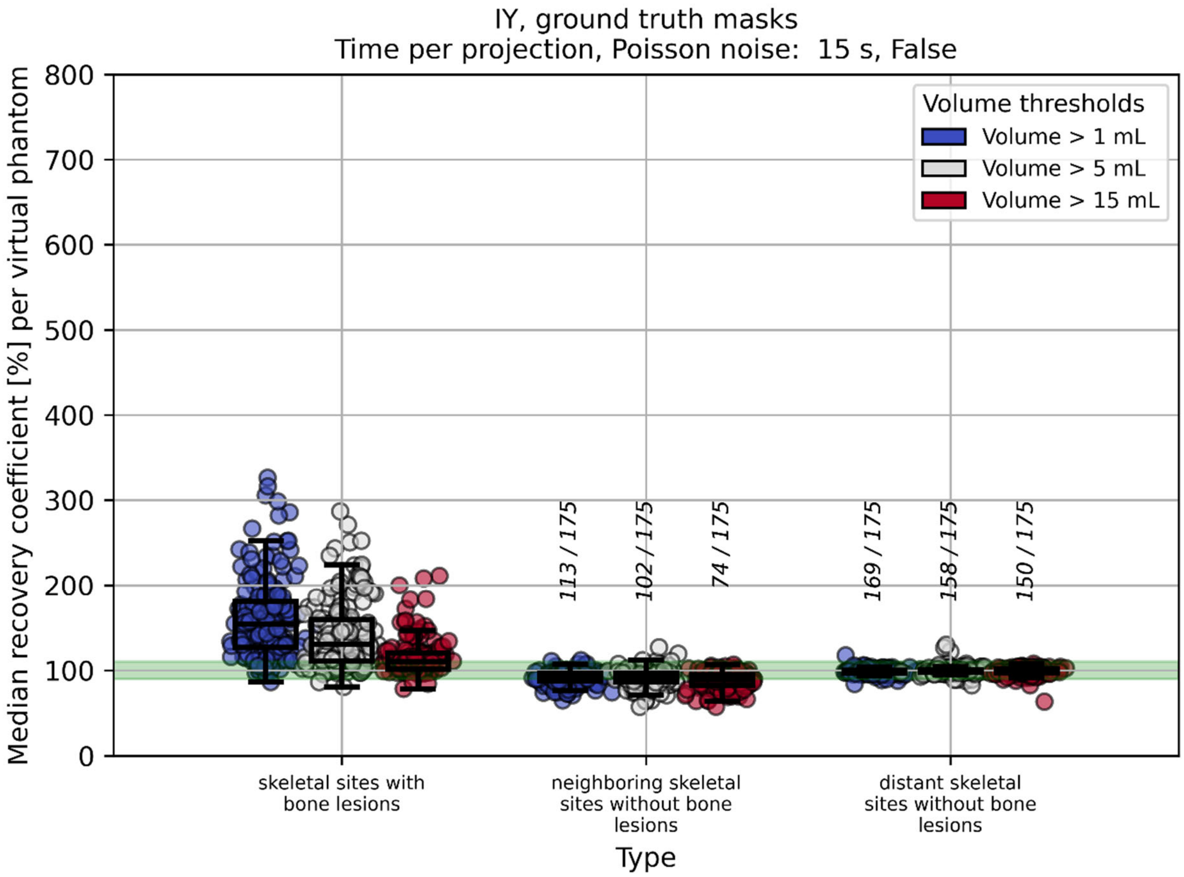

Figure S20. Median RCs per virtual phantom for methods 1-3 from Table 1, depending on the selected VOI volume threshold, shown for 15 s per projection without Poisson noise and images processed with IY using ground truth masks.

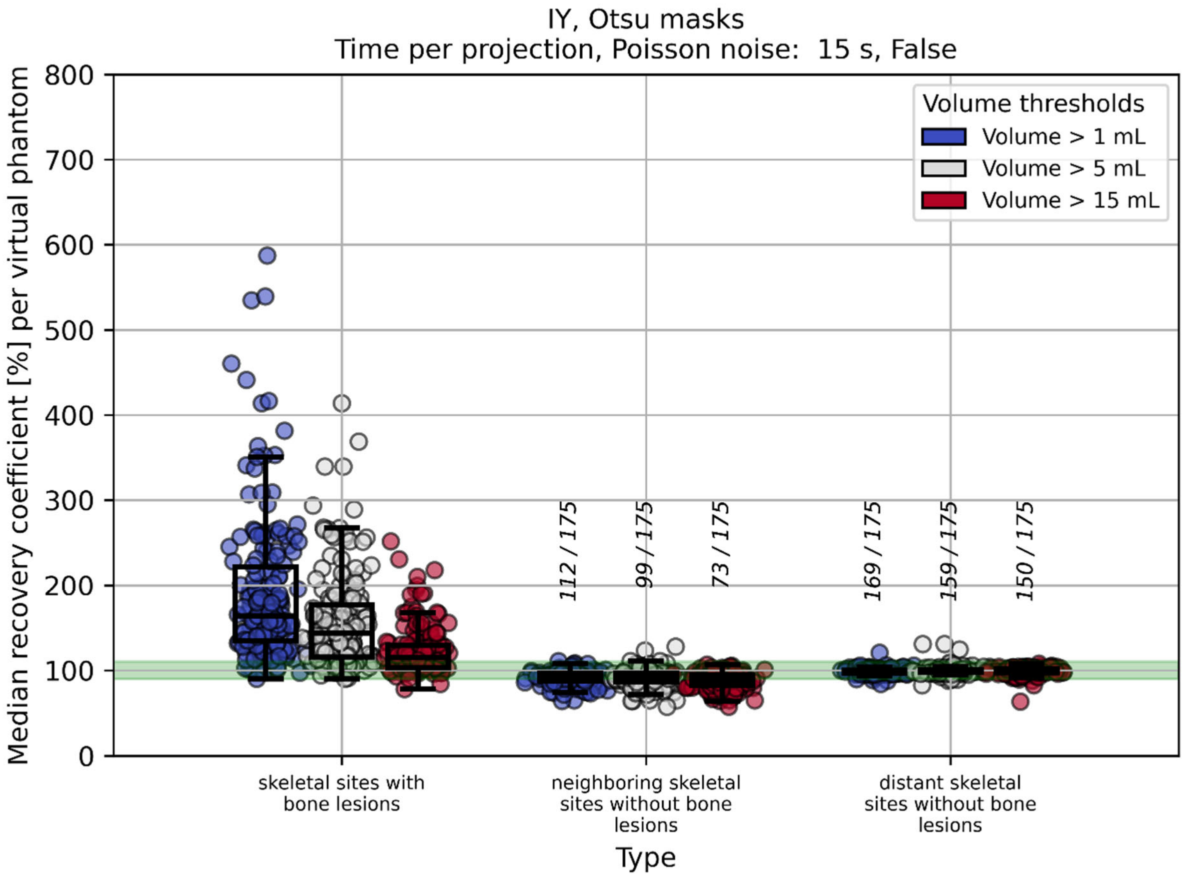

Figure S21. Median RCs per virtual phantom for methods 1-3 from Table 1, depending on the selected VOI volume threshold, shown for 15 s per projection without Poisson noise and images processed with IY using Otsu-derived masks.

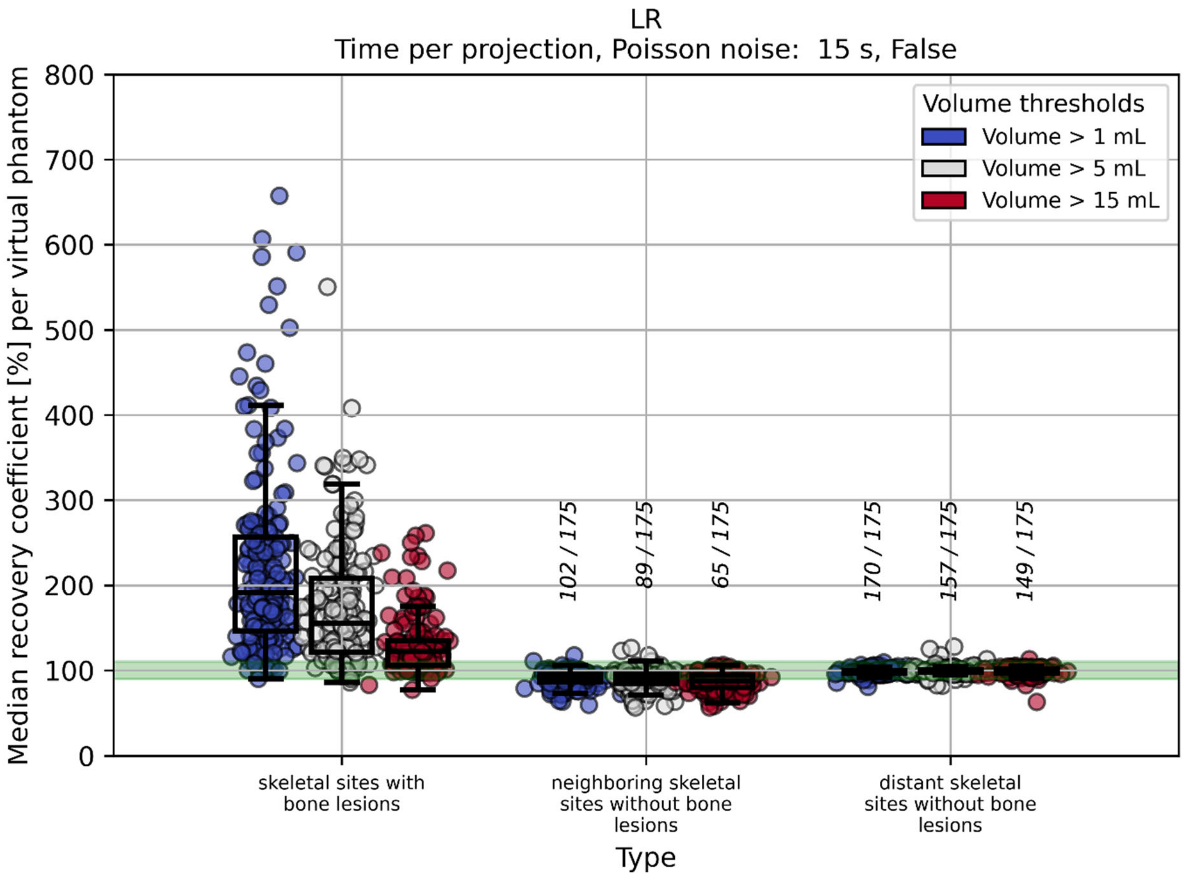

Figure S22. Median RCs per virtual phantom for methods 1-3 from Table 1, depending on the selected VOI volume threshold, shown for 15 s per projection without Poisson noise and images processed with LR.

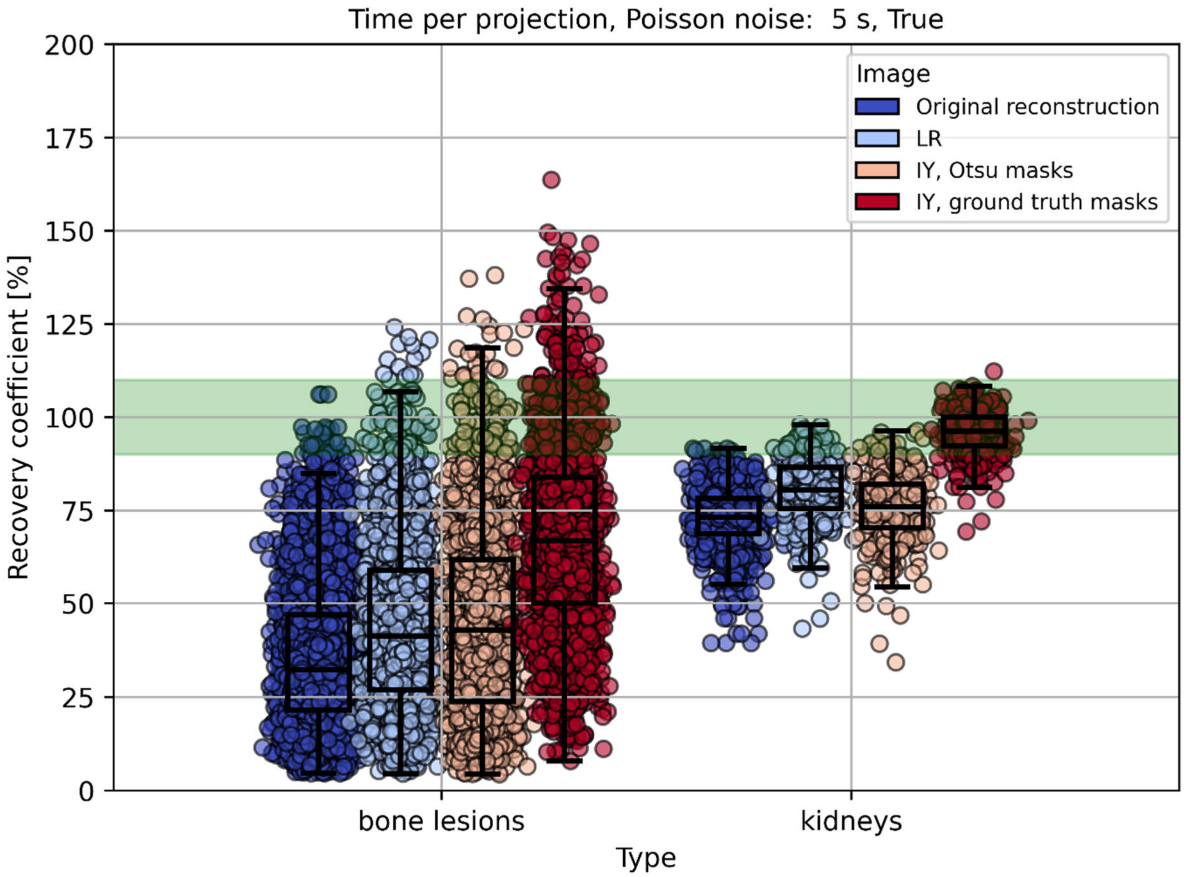

Figure S23. RCs estimated in the kidneys and bone lesions for all virtual patient phantoms. The minimum analyzed bone lesion volume is 1 mL.

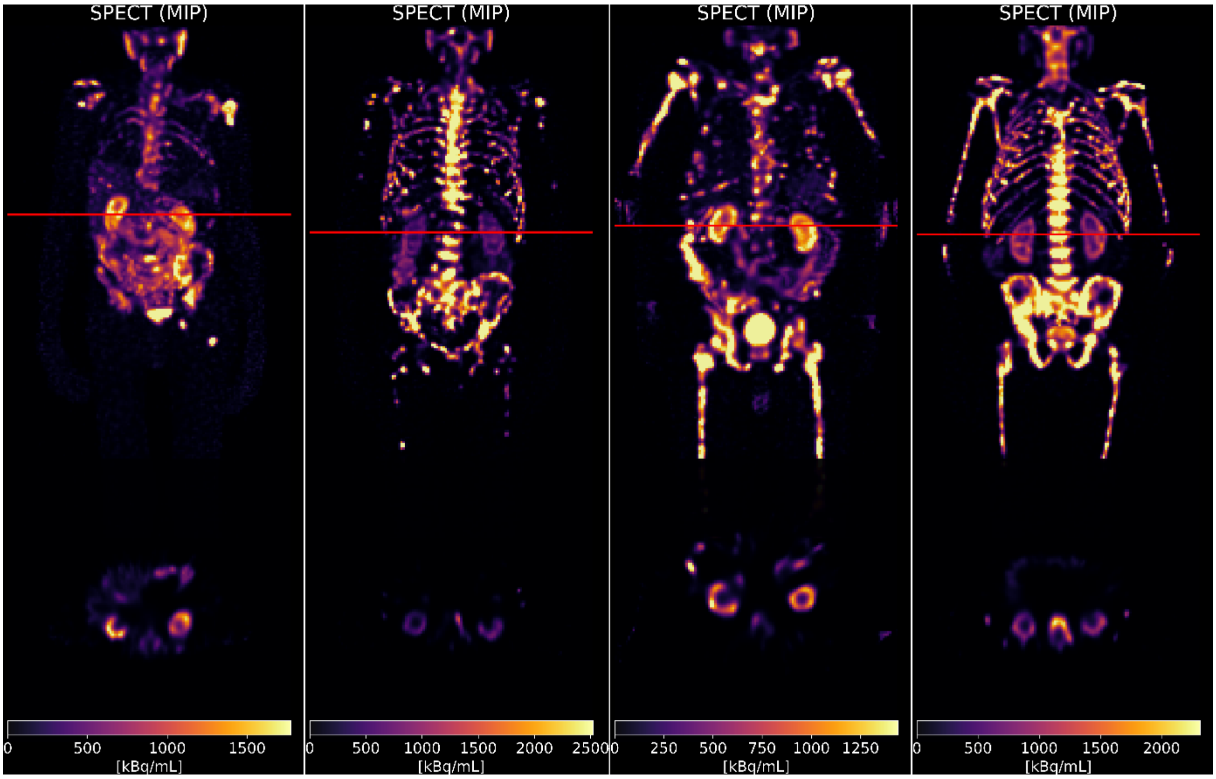

Figure S24. First-cycle SPECT images at 24 h p.i. for patients with patient IDs 2, 5, 9 and 14 (from left to right).

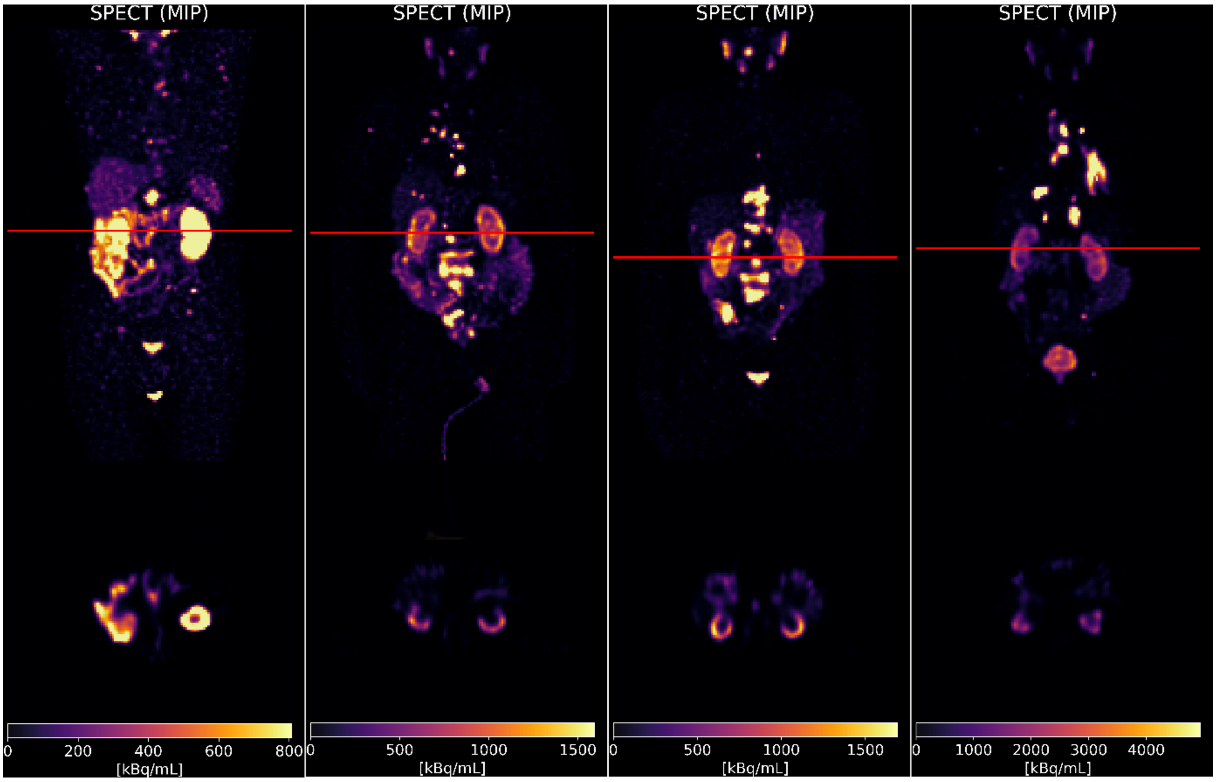

Figure S25. First-cycle SPECT images at 24 h p.i. for patients with patient IDs 1, 3, 4 and 6 (from left to right).

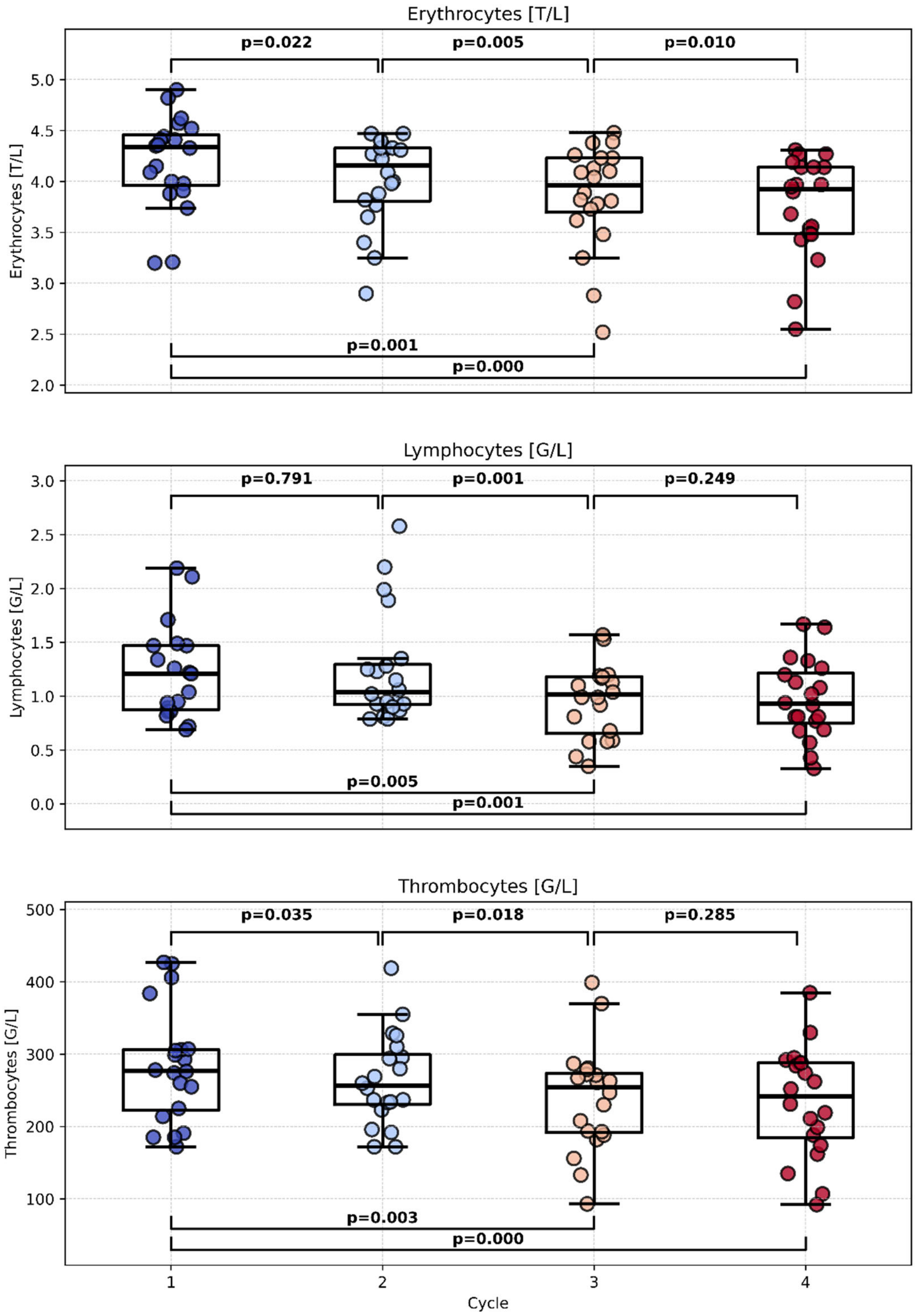

Figure S26. Blood levels of erythrocytes, lymphocytes, and thrombocytes prior to each injection across four therapy cycles. Units: G/L = 10^9 cells per liter; T/L = 10^{12} cells per liter. Paired Wilcoxon signed-rank tests with a one-sided hypothesis ("greater") were performed to assess whether blood parameter values at earlier cycles were significantly higher than those at later cycles. p-values are annotated accordingly.

Table S1. Estimated median RCs across all virtual phantoms for methods 1-3 from Table 1, including the influence of spill-over reduction, time per projection, Poisson noise and VOI volume threshold. Values for the default clinical regime are shown in bold.

Time per projection	Poisson noise	VOI volume threshold [mL]	Median RCs per virtual phantom [%] (median [min, max])											
			Skeletal sites with bone lesions				Neighb. skeletal sites without bone lesions				Distant skeletal sites without bone lesions			
			Original recon.	LR	IY, Otsu masks	IY, ground	Original recon.	LR	IY, Otsu masks	IY, ground	Original recon.	LR	IY, Otsu masks	IY, ground
**5**	**Yes**	**1**	**225 [106, 1015]**	**201 [105, 1018]**	**177 [94, 883]**	**162 [104, 352]**	**105 [72, 163]**	**103 [66, 168]**	**105 [72, 163]**	**105 [72, 163]**	**107 [77, 130]**	**107 [71, 131]**	**107 [77, 130]**	**107 [77, 130]**
15	Yes	1	228 [100, 921]	194 [94, 883]	173 [93, 653]	157 [93, 329]	98 [73, 136]	96 [61, 133]	97 [69, 136]	97 [72, 134]	103 [90, 115]	102 [88, 119]	103 [90, 115]	103 [90, 115]
15	No	1	223 [102, 921]	191 [90, 872]	164 [90, 587]	154 [86, 326]	93 [65, 119]	92 [60, 118]	93 [64, 111]	93 [65, 112]	99 [85, 121]	98 [81, 109]	99 [84, 121]	99 [84, 118]
**5**	**Yes**	**5**	**178 [92, 665]**	**163 [78, 519]**	**148 [85, 394]**	**142 [91, 279]**	**106 [72, 156]**	**104 [65, 157]**	**105 [72, 156]**	**105 [72, 150]**	**109 [71, 150]**	**108 [65, 155]**	**109 [71, 150]**	**109 [71, 149]**
15	Yes	5	178 [100, 620]	166 [90, 520]	151 [93, 334]	138 [92, 283]	97 [63, 136]	95 [61, 133]	97 [63, 136]	97 [63, 134]	104 [84, 136]	104 [76, 128]	104 [84, 136]	104 [83, 133]
15	No	5	172 [92, 654]	155 [86, 551]	144 [90, 414]	131 [81, 287]	92 [58, 128]	91 [57, 126]	92 [58, 128]	92 [58, 127]	99 [83, 132]	99 [82, 128]	99 [83, 131]	99 [83, 130]
**5**	**Yes**	**15**	**141 [91, 354]**	**133 [86, 302]**	**128 [72, 263]**	**123 [88, 224]**	**105 [50, 137]**	**104 [44, 140]**	**105 [50, 137]**	**105 [50, 137]**	**109 [97, 159]**	**109 [95, 164]**	**109 [97, 159]**	**109 [97, 159]**
15	Yes	15	137 [95, 329]	126 [90, 284]	122 [91, 256]	116 [92, 230]	96 [63, 120]	95 [62, 123]	96 [63, 120]	96 [63, 120]	104 [81, 115]	104 [81, 117]	104 [81, 115]	104 [81, 115]
15	No	15	131 [82, 316]	122 [77, 261]	115 [78, 252]	110 [79, 211]	89 [58, 107]	88 [57, 107]	89 [58, 107]	89 [58, 107]	99 [64, 108]	99 [63, 113]	99 [64, 108]	99 [64, 108]

References

1. Wasserthal J, Breit HC, Meyer MT, Pradella M, Hinck D, Sauter AW, et al. TotalSegmentator: Robust Segmentation of 104 Anatomic Structures in CT Images. Radiol Artif Intell. 2023;5(5):e230024.
2. Otsu N. A Threshold Selection Method from Gray-Level Histograms. IEEE Trans Syst Man Cybern. 1979;9(1):62-6.
3. Ljungberg M, Strand S-E. A Monte Carlo program for the simulation of scintillation camera characteristics. Comput Methods Programs Biomed. 1989;29(4):257-72.
4. Hindorf C, Glatting G, Chiesa C, Linden O, Flux G, Committee ED. EANM Dosimetry Committee guidelines for bone marrow and whole-body dosimetry. Eur J Nucl Med Mol Imaging. 2010;37(6):1238-50.
5. Stabin MG, Sparks RB, Crowe E. OLINDA/EXM: The Second-Generation Personal Computer Software for Internal Dose Assessment in Nuclear Medicine. J Nucl Med. 2005;46(6):1023-7.
